# WASP: a software package for correctly characterizing the topological development of ribbon structures

**DOI:** 10.1038/s41598-020-80851-8

**Published:** 2021-01-15

**Authors:** Zachary Sierzega, Jeff Wereszczynski, Chris Prior

**Affiliations:** 1 Department of Physics and The Center for Molecular Study of Condensed Soft Matter, Illinois Institute of Technology, Chicago, IL 60616 USA; 2 Mathematical Sciences, Durham University, Durham, DH1 3LE UK; 3 Present Address: Department of Physics, University of Colorado, Boulder, CO 80309 USA

**Keywords:** Applied mathematics, Software, Computational biophysics, Molecular biophysics, Single-molecule biophysics, Condensed-matter physics

## Abstract

We introduce the Writhe Application Software Package (WASP) which can be used to characterisze the topology of ribbon structures, the underlying mathematical model of DNA, Biopolymers, superfluid vorticies, elastic ropes and magnetic flux ropes. This characterization is achieved by the general twist–writhe decomposition of both open and closed ribbons, in particular through a quantity termed the polar writhe. We demonstrate how this decomposition is far more natural and straightforward than artificial closure methods commonly utilized in DNA modelling. In particular, we demonstrate how the decomposition of the polar writhe into local and non-local components distinctly characterizes the local helical structure and knotting/linking of the ribbon. This decomposition provides additional information not given by alternative approaches. As example applications, the WASP routines are used to characterise the evolving topology (writhe) of DNA minicircle and open ended plectoneme formation magnetic/optical tweezer simulations, and it is shown that the decomponsition into local and non-local components is particularly important for the detection of plectonemes. Finally it is demonstrated that a number of well known alternative writhe expressions are actually simplifications of the polar writhe measure.

## Introduction

Quantifying the varying and complex geometries of three dimensional curves is an important task in many fields. For example, flexible biomolecular structures such as polymer chains, DNA helices, and chromatin fibers adopt a wide range of conformations when exposed to different solvent/cosolute environments and external forces and torques^1–3^. Characterizing the evolving morphology of these chains poses significant mathematical challenges that naturally draw on the fields of topology and differential geometry.

**Figure 1:**
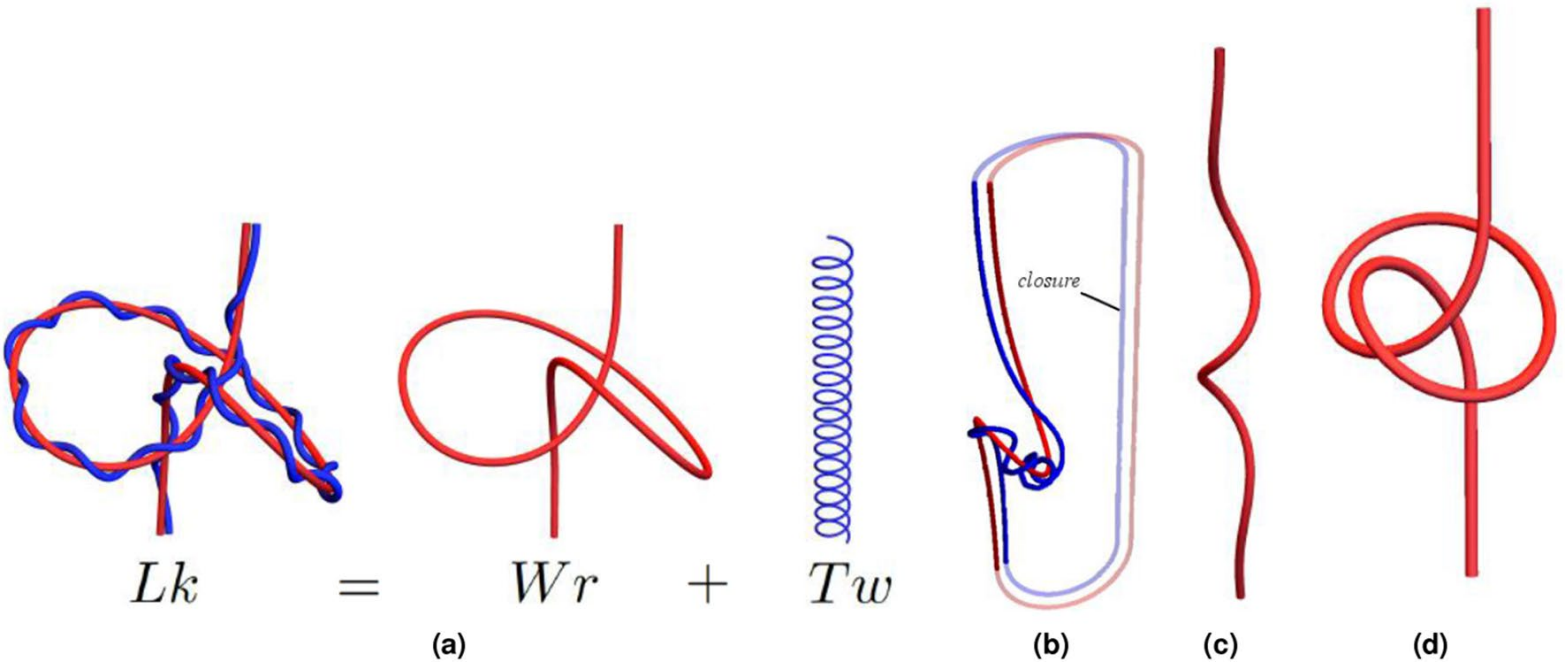
Illustrations of concepts discussed in the introduction. (**a**) is an illustration of the meaning of the $Wr+Tw$ decomposition. The left figure is a ribbon structure composed of an axis curve (red) and a second curve wrapping around this axis (blue). The linking of these two curves (invariant if the ends of the ribbon are fixed) can be decomposed into the self linking of the ribbon’s axis (*Wr*) and the total rotation of the second curve around the axial direction of the first *Tw*. (**b**) indicates an artificial extension of the ribbon (a closure). (**c**,**d**) illustrate what is meant by local and non-local writhing. The curve in (**c**) coils helically at its centre; the local writhe measures this helical coiling along the curve’s length. The curve in (**d**) is knotted/self entangled, that is to say distinct sections of the curve wrap around each other. This is non-local writhing.

One of the most useful metrics for quantifying large scale structural changes in these complexes is writhe. The writhe is a global geometric quantity commonly used to characterize the conformational variety of circular DNA structures^4^. Its utility relates to the fact that, for ribbon structures (see Fig. 1a) which can be used to model structures ranging from DNA molecules to magnetic flux ropes, it forms part of the invariant sum:

1 $$\begin{aligned} Lk = Wr+Tw. \end{aligned}$$

Here *Lk* is the linking number, a topological measure of the entwined nature of the two edges of the ribbon that is invariant under any change in shape which forbids their crossing (i.e it categorizes the ribbon’s entanglement). The writhe *Wr* represents the contribution to *Lk* from the self linking of the axis curve on itself (Fig. 1a). The twisting *Tw* is a measure of the rotation of the ribbon about its axis^5^ (Fig. 1a). As an example of its use, *Lk* can be used as a fixed model constraint on the number of DNA sequence repeats. The constraint is then applied to an energy model of the DNA backbone which often has the twisting *Tw* as an elastic energy component^6–11^. The writhe can then be used to constrain the allowed global (axial) shape of the molecule. In DNA models writhe measures supercoiling.

The most commonly known version of the link–twist–writhe relationship, Eq. (1), is for closed ribbons/curves such as DNA minicircles. The relationship given in Eq. (1) was originally derived by Călugăreanu^12–14^ and was popularised (but not derived) for a biological audience by Fuller^5,15^. However, the theorem as originally derived is not applicable to open-ended ribbons such as those shown in Fig. 1a. This is because (i) the linking (*Lk*, as defined in that version of the theorem) is not an invariant for open-ended ribbon structures^16^ and (ii) there is no evidence that the equality in (1) holds if the ribbon is not closed. Therefore, it cannot constrain the interplay between internal twisting (*Tw*) and global self entanglement of the ribbon’s axis (*Wr*). Many interesting target applications which can be modelled by ribbons are open structures such as DNA molecules subjected to optical tweezer experiments and chromatin fibers.

To overcome this problem, Fuller originally suggested that one could extend the closed ribbon theorem to open ribbons by artificially extending the structure^15^ as shown in Fig. 1b. This approach has been popular in the field of DNA modelling^7,8,17–21^ and elastic tube applications^22–24^. It is complicated by the fact that the closure will generally contribute to both the writhing and the linking of the composite and hence the extraction of clear geometrical insight from the calculated quantities cannot be consistently achieved^16,25,26^. Alternative approaches have been formulated which approximate the writhing value^7,8,15,17,23,27,28^, however, these will in general not charcterize the writhing^16,25^ and hence cannot consistently be used to constrain the topology of the ribbon. Finally, some authors have simply chosen to utilize the definition of the original closed formula without a closure. In this case the *Lk* measure is no longer a topological invariant and the relationship loses its fundamental topological anchoring (the sum $Tw + Wr$ is no longer fixed).

Previously, Berger and Prior^16^ introduced a version of (1) which is applicable to open-ended ribbons. It is based on a definition of *Lk* (called the net-winding) which is invariant to all deformations of the ribbon that do not permit rotation at its ends. In supercoiling ribbon models it would be equivalent to the natural linking of the DNA plus the applied over-coiling of the structure. The twisting *Tw* is the same quantity as in the closed case and a definition of the writhing (termed the polar writhe) was based on the difference of these quantities. For closed ribbons this is equivalent to the original Călugăreanu theorem, but, since the net winding is also an appropriate topological constraint for open ribbons, it has a wider range of applications than the original theorem. This framework has been regularly applied in the field of Solar physics since the original paper e.g.^29–35^ and is increasingly being applied in Elastic tube modelling^26,36,37^ as well. However, it has not been widely adopted in the biophysical community and artificial closure methods are still readily employed for problems such as characterizing DNA supercoiling.

Here, we seek to introduce polar writhe to the biophysical community and to provide a user-friendly tool for its implementation. The first aim of this article is to introduce the open-ribbon version of Eq. (1) derived in^16^ and extended to consider knotting deformations in^26^. Both papers are somewhat technical, necessarily involving mathematical proofs. In this note we illustrate the properties of the polar writhe which underpin this open decomposition though instructive examples of constrained DNA supercoiling simulations. The second aim is to demonstrate how decomposing the polar writhe into local and non-local components, which measure “spring-like extension” deformations and “buckling-type” deformations/supercoiling changes respectively (see Fig. 1c,d), can provide critical additional insight into the curve’s geometry through a series of instructive examples. Third, we demonstrate how an extension of (1) as defined in^26^ can detect transitions to knotting possible in open ribbon structures even when the ends of the ribbons are constrained from moving. Finally, we introduce the *Writhe Analysis Software Package* (WASP) which can calculate the components of Eq. (1) in both closed and open ended cases. WASP allows non-expert users to easily apply these calculations to standard curve files and is particularly geared towards usage with molecular dynamics trajectories, supporting common trajectory file types including those generated by popular MD software including AMBER and OxDNA as well as more general MD file types such as PDB files.

The paper is structured as follows. In the first section, we introduce the polar writhe through a series of instructive example calculations. This includes highlighting the local/non-local decomposition and the additional information it provides. The Methods section provides a description of the DNA simulation experiments that were performed and designed to highlight aspects of the open *Lk*–*Tw*–*Wr* decomposition described in^16,26^. This includes a description of how the algorithms in WASP function. The Results section details the analysis of these experiments and highlights how aspects of the polar writhe measure such as the local/non-local components and knot detection provide insight. In particular, we demonstrate how the non-local component of the polar writhe can serve as an indicator of plectoneme formation in both linear and circular DNA structures. These sections are aimed at providing a clear and easy to read guide to demonstrate how the WASP package can be used to provide insight into the evolving geometry of 3-D curves and ribbon structures such as those inherent in DNA structures. Additionally, in the final section we show how WASP can be used to detect knot and belt-trick type deformations, again through an instructive example. Finally, we provide a supplement which is of a much more technical nature and aimed at readers with a specific interest in ribbon topology. It details how the original closed Călugăreanu theorem (the closed ribbon *Lk*–*Tw*–*Wr*) and the Fuller writhing quantities all arise naturally as part of the framework described in^16,26^. It is not necessary to read this supplement in order to use and interpret the WASP package, but it would be necessary in order to compare it to existing writhe calculation approaches.

## Introduction to the polar writhe

In typical applications, *Lk* is a prescribed quantity (i.e. a fixed applied number of rotations of the structure in plectoneme experiments), and the twist *Tw* can be calculated as *Lk*-*Wr*. Thus, in this section, we focus on the definition of the writhing as the key quantity to be calculated. In^16,26^ the definition of writhing was given the name the *polar writhe* which we label $W_p$ to distinguish from the pre-existing closed ribbon definition (the reason for this name will be clarified shortly). In what follows, we introduce the quantity $W_p$ through a series of instructive examples.

**Figure 2:**
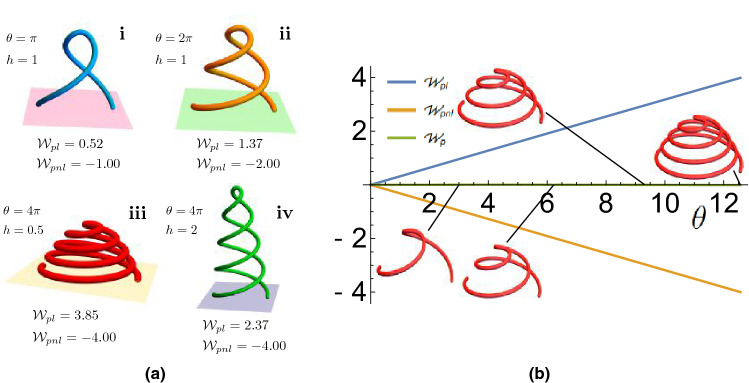
Twisted paraboloids used to highlight aspects of the polar writhe. (**a**) paraboloids with varying heights *h* and winding angles $\theta$. (**a**)(i),(ii) paraboloids with equal height but different winding exhibit differences in both $W_{pl}$ and $W_{pnl}$ as a result of large-scale rotation and increased helical density. (**a**)(iii),(iv) paraboloids with equal winding but varying height exhibit differences in $W_{pl}$ only as the extent of buckling remains constant while helical density is varied. (**b**) The polar writhe decomposition as a function of the winding angle $\theta$ for a parabola for which $W_p=0$.

### The polar writhe

The polar writhe^16^ is the sum of a local component ($W_{pl}$) and a non-local component ($W_{pnl}$):

2 $$\begin{aligned} W_{p} = W_{pl}+ W_{pnl}. \end{aligned}$$

The twisted paraboloids shown in Fig. 2a provide an intuitive example of the behaviors tracked by these distinct components. The parabolas are described by the following formula:

3 $$\begin{aligned} \mathbf{x}(t)&= \left( (t-1/2)\cos \left( \frac{\theta z(t)}{h}\right) ,(t-1/2)\sin \left( \frac{\theta z(t)}{h}\right) ,z(t) \right) ,\nonumber \\ z(t)&=4 h t(1-t). \end{aligned}$$

The parameter $\theta$, the winding angle, determines the number of rotations applied to the parabola (c.f. Fig. 2ai,ii). The height parameter *h* determines the relative stretching of this coil (c.f. Fig. 2aiii,iv). *For this curve* $W_{pnl}=-\theta /2\pi$. The factor of $2\pi$ indicates that $W_{pnl}$ measures the number of full rotations. The sign is due to the orientation of the curve as we shall explain shortly.

In the top row of Fig. 2a, *h* is fixed. In (a), $\theta = \pi$, resulting in a single loop around the middle of the paraboloid. In this case $W_{pnl} = -1$. In b there is an additional looping and $W_{pnl}=-2$. The local component, which measures the local helical density of the paraboloid, is increased from (a)(i) to (a)(ii) due to additional winding at fixed height. In the bottom row we fix the winding ($W_{pnl}=-4$) but vary the curve’s height *h* to highlight the effect on $W_{pl}$. As the height of paraboloid is increased from (a)(iii) to (a)(iv), the overall winding (and hence the non-local writhe) is unchanged. However, as the coil becomes increasingly less tightly wound, the local writhing decreases as its helical density is decreased. Note that in these cases the two components are generally of opposite sign, however, this is *not* a general property of the pair $(W_{pl},W_{pnl})$. As shown in^16^, if one chooses $h\approx 0.37$ then $W_p\approx 0$ irrespective of the choice of $\theta$ (see Fig. 2aii). We see the added value of the local/non-local decomposition providing information about the increasingly tight helical coiling which is missing from the total sum.

#### Curve splitting

**Figure 3:**
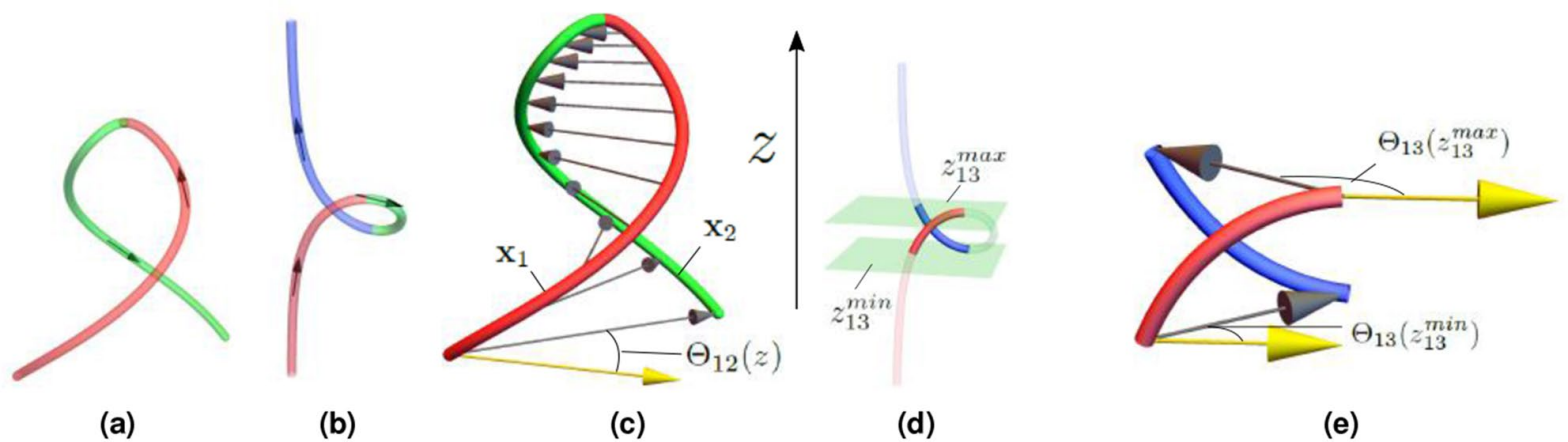
Illustrations of the non-local writhe calculation. (**a**,**b**) are curves split by their turning points. Their orientations are shown by arrows. (**a**) The parabola’s turning point is at its peak and the two sections (red and green) which are partitioned by this turning point are shown. (**b**) A looped curved which could represent the beginning of plectoneme formation. It has two turning points at the top and bottom of the loop. The curve is split into three sections, red, green and blue respectively, by these turning points. (**c**) The two sections of the parabola $\mathbf{x}_1$ and $\mathbf{x}_2$ share a common mutual height $z\in [0,h]$. At each height a vector is drawn from $\mathbf{x}_1$ to $\mathbf{x}_2$. As indicated, they make an angle $\Theta _{12}(z)$ with respect to a fixed direction. The non-local polar writhe measures the rotation of this angle. (**d**) Subsections $\mathbf{x}_1$ and $\mathbf{x}_3$ of the curve shown in (**b**) which share a mutual *z* range between two planes $z=z_{13}^{min}$ and $z_{13}^{max}$. These subsections are shown in bold coloring. (**e**) The $W_{pnl}$ calculations for the mutual subsections shown in (**d**). The difference in the angles $\Theta _{13}(z_{13}^{min})$ and $\Theta _{13}(z_{13}^{max})$ which characterises the non-local writhing contribution from these two sections is depicted.

The idea behind the polar writhe calculation is that we split the curve into sections sharing a mutual vertical height. If we have a Cartesian coordinate system (*x*, *y*, *z*) where *z* is the height, then we split the curve at *turning points* for which the curve changes direction from upward to downward pointing: $\hat{z}\cdot \text {d}\mathbf{x}/\text {d}z=0$. Examples of the parabolas (1 turning point, 2 sections) and a locally looped structure (2 turning points, 3 sections) are shown in Fig. 3a,b. In general, we label the subsections *i* of the curve that span heights $z \in [z_i^{min},z_i^{max}]$ as $\mathbf{x}_i$. The local writhe quantifies the coiled geometry of each individual subsection and the non-local writhe quantifies the mutual winding of all distinct pairs of sections.

#### Local polar writhe

If the unit tangent vector of the curve $\mathbf{x}$ is $\mathbf{T}= \text {d}\mathbf{x}/\text {d}{s}$ (*s* being the arclength of the curve), then:

4 $$\begin{aligned} W_{pl}(\mathbf{x})=\frac{1}{2\pi }\sum _{i=1}^{n}\int _{z_i^{min}}^{z_i^{max}}\frac{\hat{z}\cdot \mathbf{T}_{i}\times \frac{\text {d}\mathbf{T}_{i}}{\text {d}z}}{1+|\hat{z}\cdot \mathbf{T}_i|}\,\text {d}z \end{aligned}$$

The numerator represents the rotation of the unit tangent vector around the vertical direction. It is positive if the curve coils in a right-handed fashion and negative if left-handed. The denominator indicates (i) the rotation is given more weight if the curve is rotating relatively tightly (as with a small *h* parabola) and (ii) the modulus sign means that the orientation would be the same for a curve whether it points up or down, i.e. it only depends on the local helical chirality of the curve. The fact that this rotation is measured around the $\hat{z}$ direction, which on a unit sphere of directions is the north pole, and that $W_{pl}$ can be interpreted as an area on the unit sphere bound by the curve $\mathbf{T}$ and the pole is the reason for the name the polar writhe (see^16^). However, this is not a crucial point in what follows and we do not mention it any further.

#### Non-local polar writhe

Consider the parabola example. The curve is separated into sections $\mathbf{x}_1$ and $\mathbf{x}_2$ (as shown in Fig. 3c). We define an angle $\Theta _{12}(z)$ which is the angle made by the vector joining section $\mathbf{x}_1$ to $\mathbf{x}_2$ at a fixed height *z* as shown in Fig. 3c. $W_{pnl}$ measures the total number of rotations of this angle:

5 $$\begin{aligned} \frac{\Delta \Theta _{12}}{2\pi }= \frac{1}{2\pi }\int _{0}^{h}\frac{\text {d}\Theta _{12}}{\text {d}z}\text {d}z. \end{aligned}$$

To ensure that $W_{p}$ forms part of an invariant sum with the twisting, the orientation of the curve sections is accounted for by multiplying by an indicator function $\sigma _{i}$ with $\sigma _i=+1$ if the curve section $\mathbf{x}_i$ is moving upwards and $\sigma _i=-1$ if it is moving downwards. Additionally, the calculation is counted twice. This is necessary to form part of the invariant sum $W_p+Tw$. Thus for the parabola:

6 $$\begin{aligned} W_{pnl}= 2\sigma _1\sigma _2\frac{\Delta \Theta _{12}}{2\pi } = -\frac{\theta }{\pi }. \end{aligned}$$

For the looped curve shown in Fig. 3b we follow a similar procedure for the pairs $(\mathbf{x}_1,\mathbf{x}_2)$, $(\mathbf{x}_1,\mathbf{x}_3)$ and $(\mathbf{x}_2,\mathbf{x}_3)$. The only extra complication is that the curve sections do not share the same mutual *z* ranges and the winding is only measured for subsections of the curve which share the same mutual *z* range $z\in [z_{ij}^{min},z_{ij}^{max}]$ as indicated in Fig. 3d. The non-local writhe for the pair $(\mathbf{x}_1,\mathbf{x}_3)$ would be:

7 $$\begin{aligned} W_{pnl}(\mathbf{x}_1,\mathbf{x}_3)= 2\sigma _1\sigma _3\frac{\Delta \Theta _{13}}{2\pi } = \frac{ \Theta _{13}(z_{13}^{max})-\Theta _{13}(z_{13}^{min})}{\pi }, \end{aligned}$$

as indicated in Fig. 3e. Note that the product of $\sigma _1\sigma _3$ here is positive as both sections have the same vertical orientation. The total $W_{pnl}$ of this curve would be:

8 $$\begin{aligned} W_{pnl}(\mathbf{x})=2\sigma _1\sigma _2\frac{\Delta \Theta _{12}}{2\pi } +2\sigma _2\sigma _3\frac{\Delta \Theta _{23}}{2\pi }+2\sigma _1\sigma _3\frac{\Delta \Theta _{13}}{2\pi }. \end{aligned}$$

In general, $W_{pnl}$ is just the mutual winding of all subsections of the curve which share a mutual height range. In the general case where the curve $\mathbf{x}_i$ has *n* sections (and $n-1$ turning points) the non-local writhing is calculated as:

9 $$\begin{aligned} W_{pnl}=\mathop {\sum _{i=1}^{n}\sum _{j=1}^{n}}_{i\ne j}\frac{ \sigma _{i}\sigma _{j}}{2\pi }\int _{z_{{ij}^{min}}}^{{z_{ij}}^{max}}\frac{\text {d}\Theta _{ij}(z)}{\text {d}z}\,\text {d}z. \end{aligned}$$

#### Looped local to non-local transition

**Figure 4:**
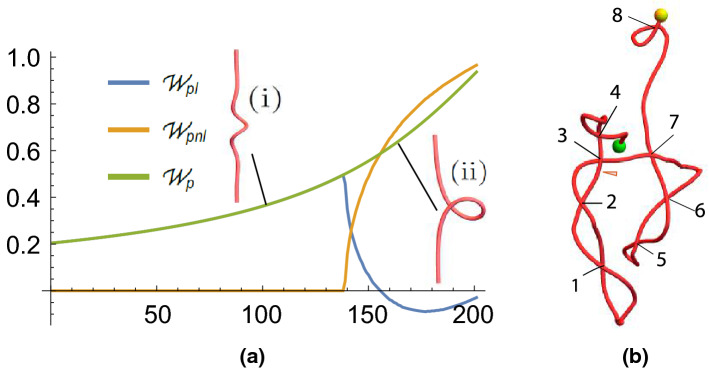
Polar writhe calculations of plectoneme geometries. (**a**) $W_p$ values of a loop forming curve deformation. (**b**) A curve with significant plectoneme structure. The individual loops present are marked.

We consider a second illustrative example: a curve transitioning from a locally helically coiled curve to a looped curve, the first part of plectoneme formation. In this case, the looped curve is the curve shown in Fig. 3b and used as an example above. This transition is described by a set of curves which are equilibria of elastic rod models:

10 $$\begin{aligned} R(s,\tau )&= \frac{2}{1+\tau ^2}\text {sech}(s),\quad \Phi (s,\tau ) =\tau s -\pi /2,\nonumber \\ z(s,\tau )&= s-\frac{2}{1+\tau ^2}\text {tanh}(s),\quad \mathbf{x}(s,\tau )= \left( R\cos \Phi ,R\sin \Phi ,z\right) . \end{aligned}$$

Here $s\in [-5,5]$ is the curve’s arclength and $\tau$ is a parameter which acts to twist the tube as it is decreased, here from 3 to 0.2. As the tube is twisted, its axis transitions from a curve with a helical perversion around its centre, see Fig. 4(i), to a looped curve indicative of the beginning of plectoneme formation as shown in Fig. 4(ii). One can obtain this behaviour by steadily twisting a cable under tension; that they are are equilibria of an elastic tube model is shown in^38^, and thus they represent the deterministic counterpart of a worm-like ribbon model e.g.^7,8^. In Fig. 4 we see its $W_p$ values as $\tau$ is decreased from 3 to 0.2 in 200 evenly spaced steps. The polar writhe $W_p$ is seen to steadily increase as the curve becomes at first increasingly helically coiled at its centre and then increasingly looped. For curves 1–138 all of the contribution to $W_p$ is local due to the helical kinking of the curve around its centre ($W_{pnl}$ = 0). After this, the loop begins to form and the curve develops non-local writhing ($W_{pnl}>0$). What is interesting is the dramatic inter-conversion of local and non-local writhing which occurs whilst the sum changes smoothly through this transition, indicating that the heilical coiling becomes distorted to form the loop. Again we see the local/non-local decomposition providing significant extra information which characterizes the geometrical development of the curve.

#### Non-local writhing detecting plectoneme formation

An example of a highly plectonemed curve is shown Fig. 4. Marked on the curve are 8 clear crossings indicating looped geometry. Since we have seen in the parabola example (Fig. 2) that loops typically have a $W_{pnl}$ value of $\pm 1$, we should expect the non-local writhing of this curve to have a magnitude of around 8. In fact, for this curve, $W_p=8.88$, $W_{pnl}=8.55$, and $W_{pl}=0.33$ (all to 3.s.f). Thus, we can infer that for highly plectonemed curves, $W_p$ will be largely dominated by non-local writhing and will roughly count the number of loops of the plectoneme.

Finally, for the interested reader, an additional set of example calculations including local/non-local decompositions of example curves can be found in Chapter 4 of^39^ and^16^ and examples of elastic rod equilibria can be found in in chapter 6 of^26^. Shortly, we will turn our attention to calculating this quantity for the output of numerical supercoiling experiments.

### Self crossing detection

Both the linking *Lk* (the net winding) and the polar writhe $W_p$ change by a a value of $\pm 2$ if the ribbon intersects itself (the twist changes continually). This would, for example, be relevant in DNA models for topoisomerase action. In^26,36^, this fact was used to detect whether solution branches for an elastic tube model were physically valid. A series of evolving elastic equilibria were obtained using a continuation method and when the values of $W_p$ jumped by $\pm 2$ the equilibrium path could be automatically ruled out as not physically accessible (as elastic tubes cannot self intersect). Of course in DNA modelling we typically expect this to be impossible due to localized repulsion of the polynucleotide chains.

## Methods

### Numerically simulating DNA tweezer experiments

Plectonemic DNA structures were generated via coarse-grained molecular dynamics simulations of magnetic/optical tweezer experiments. 600 base-pair linear DNA helices were constructed with three different net-windings: $Lk = 40 \;\;(\approx 24^{\circ } \text {per\, turn})$ (underlinked) , $Lk = 60 \;\;(\approx 36^{\circ }\, \text {per\, turn)}$ (torsionally relaxed), and $Lk = 80 \;\;(\approx 48^{\circ } \text {per \,turn)}$ (over-linked). Their evolution was simulated using the oxDNA2 model^40^. To simulate tweezers experiments, each helix was first aligned such that its axis was along the $\hat{z}$ direction. Anchor restraints were placed on the bottom five base-pairs of each helix to permanently fix their position and harmonic restraints were applied to the top five base-pairs of each helix to allow the respective base pairs to move only in the $\hat{z}$ direction and also to prevent the DNA from shedding its winding by simply untwisting. In addition to these restraints, a range of extension forces (0pN, 2pN, 4pN, 6pN, 8pN) were applied to each helix in separate simulations by pulling the top five basepairs in the $+\hat{z}$ direction. Each helix was simulated for a total production run of $1.2 {\times}10^8$ timesteps using the oxDNA2 model with a salt concentration of $0.5\;$ M. We only detail three specific cases below for brevity. These cases are chosen as they highlight the critical characteristics of the polar writhe measure as applied to these numerical supercoiling experiments.

### Atomistic molecular dynamics simulations of supercoiled DNA minicircles

108 base-pair minicircles were constructed with $Lk = 14$ ($\Delta Lk = +4$) to promote conformational variety/structural buckling predicted by previous experimental/simulation explorations of minicircle dynamics^4,41^. Two base-pair sequences, (AA)$_{27}$(AT)$_{27}$(AA)$_{27}$(AT)$_{27}$ and (GG)$_{27}$(GC)$_{27}$(GG)$_{27}$(GC)$_{27}$, were explored as well. Simulations were performed in explicit solvent using AMBER with a modified DNA BSC1 force field to correct for the circularized DNA and Smith and Dang ion modifications for Na and Cl ions^42–44^. DNA minicircles were solvated in a TIP3P rectangular water box with a 15.0 Å buffer. Following solvation, the appropriate cosolute (spermine) was placed along with the DNA within the water box and the entire system was neutralized with Na+ ions. Initial minimization was performed on the cosolute and water molecules with a 10 kcal/mol restraint placed on the DNA residues. After the initial minimization, restraints were removed from the DNA and the entire system was allowed to minimize. Following the minimization phase, restraints were reapplied to the DNA residues and the system was heated from 100 to 300 K. Consequent to heating, restraints were progressively removed from the DNA residues over a period of $\approx$ 4 ns. MD was performed without restraints on the equilibrated system for 100ns for each trial in the NVT ensemble. The number of spermine molecules in each simulation varied between trials to explore different ranges of cosolute concentration.

### Obtaining a DNA axis

To calculate $W_p$, it is first necessary to generate an axis for the DNA molecule. In order to properly characterize the geometry of a given DNA structure, it is important to generate an axis curve in a manner such that its total curvature is minimized. Failure to do so may result in the misrepresentation of the DNA geometry due to excess local writhe introduced to the axis curve as a result of the helical nature of the DNA backbone strands. Simplistic axis curve formulations such as the axis curve obtained by taking the midpoint of the C1’ atoms located on opposite sides of each base-pair along the helix result in coiled axes that exhibit the same helical periodicity as their encompassing backbone strands. To avoid such misrepresentation, the WASP package uses an implementation of the WrLINE axis curve method developed by Sutthibutpong, Harris and Noy^45^ which effectively smooths the contour of the axis curve and eliminates the unwanted “coiling” and helical periodicity exhibited by alternative methods that generate unwanted excess local writhe. Full details regarding the WrLINE formulation can be found in^45^, but key features of the formulation are summarized below maintaining similar notation as in^45^.

**Figure 5:**
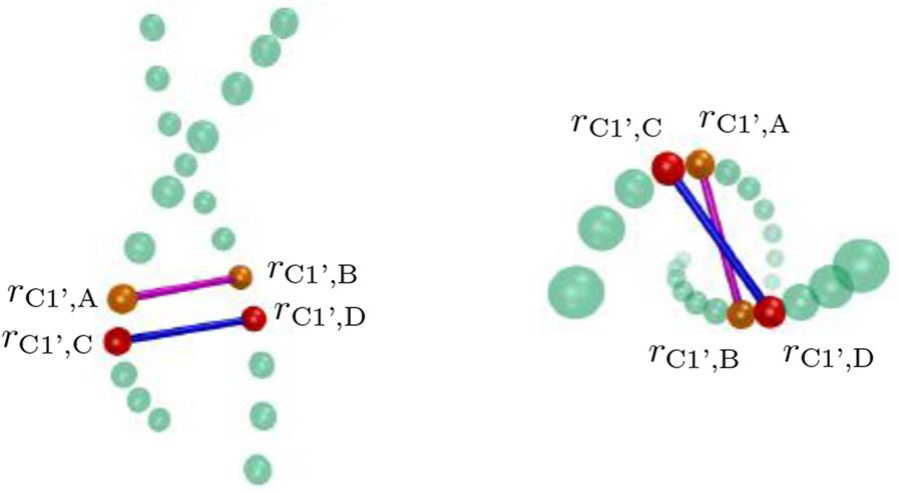
Left: Side perspective of backbone atoms along a helical fragment of DNA. Beads **r** represent C1’ atoms along the DNA backbone. Right: Top view of backbone atoms along the helical fragment.

Unlike a basic midpoint method in which an axis is obtained by simply calculating the midpoint $m_i$ of the pair of C1’ atoms ($r_{\text {C1',A}}$ & $r_{\text {C1',B}}$—see Fig. 5) on opposite sides of a corresponding basepair *j*:

11 $$\begin{aligned} m_j=\frac{r_{\text {C1',A}} + r_{\text {C1',B}}}{2} \end{aligned}$$

for each base-pair along the DNA helix, the midpoint $r_i$ of each “dinucleotide” (2 base-pair) step along the DNA helix consisting of the two pairs of C1’ atoms on opposing sides of a set of two consecutive base-pairs *j* & $j+1$ on the helix is calculated as:

12 $$\begin{aligned} r_i = \frac{r_{\text {C1',A}} + r_{\text {C1',B}} + r_{\text {C1',C}} + r_{\text {C1',D}}}{4}. \end{aligned}$$

The remainder of the WrLINE method can then be roughly summarized by the following procedure:

1. Given a dinucleotide midpoint $r_i$, calculate the base-pair step twist $\theta _{i}$ from the dinucleotide step corresponding to $r_i$
2. Determine the number of base-pairs (2*m*) required to complete a full helical turn around the base-pair step corresponding to $r_i$ (i.e. *m* base-pairs above $r_i$ and *m* base-pairs below $r_i$).
3. Calculate a weighting factor *w* related to the sum of the base-pair twists $\theta _{i-1}, \theta _{i+1},\theta _{i-2}, \theta _{i+2} \;...$ throughout the helical turn around $r_i$.
4. Use $r_i$, *m*, and *w* to determine a point on the axis curve $h_i$.

The above procedure is then repeated for each midpoint $r_i$ to obtain a full set of axis points $h_i$ that represent the DNA helical axis. Note that the summary of the WrLINE method provided above has been included solely for the purpose of familiarizing the reader with the axis curve method utilized in WASP as well as to highlight the intricacies of determining a proper helical axis. The reader is strongly encouraged to refer to^45^ for a comprehensive treatment of the WrLINE method.

It is important to note that the WrLINE method is intended to be used primarily with closed DNA structures such as DNA minicircles. As such, the algorithm requires that there exist a full helical turn of DNA around each point $r_i$ as specified above. This requirement is problematic in the case of linear DNA helices where a full helical turn of DNA cannot exist around points $r_i$ near the ends of the helix. In order to circumvent this issue, two options are available:

1. Treat the DNA structure as though it were closed (joined at the ends) despite the fact that it is, in reality, not. This allows the WrLINE method to be utilized verbatim as outlined in^45^, but will result in a number of undesirable stray axis points generated in arbitrary positions external to the DNA structure. The number of stray axis points will be proportional to the extent of supercoiling of the helix (how many base-pairs are required to make a full helical turn) and can be safely, manually deleted via knowledge of the system/visual inspection. These stray axis points will always constitute the “ends” of the unaltered axis as they are a product of end effects produced by the WrLINE algorithm. This option is the most general, but should be utilized with extreme caution.
2. Start the WrLINE method on the first points $r_i$ such that one full helical turn of DNA can be formed around the respective points $r_i$ on each end of the DNA helix (satisfying the criteria for the calculating the $\Theta _m$ parameter^45^) as determined prior to beginning the axis curve computation.

At the time of writing, options 1 and 2 are implemented in WASP via the *deleteatoms* and *autodelete* arguments respectively. In either case, the total number of resulting axis points will be less than the total number of base-pairs when evaluating open DNA structures using the WrLINE method properly.

### Calculating $W_p$

The WrLINE algorithm yields a discrete curve $\left\{ \mathbf{x}_j\right\} _{j=1}^{n}$. The algorithm for calculating $W_p$ for this curve is as follows:

1. Split the curve into *n* sections $\mathbf{x}_i$ by locating its turning points. This is done from the set of tangent vectors $\mathbf{T}_j = \mathbf{x}_{j+1}-\mathbf{x}_{j}$ and use of tricubic interpolation.
2. For each section $\mathbf{x}_i$ calculate $W_{pl}(\mathbf{x}_i)$ using Eq. (4). The WASP package uses a modified version of Simpson’s rule^46^.
3. Identify the range of mutual overlap of all pairs of sections $(\mathbf{x}_i,\mathbf{x}_k)$. The WASP package uses a simple binary search algorithm.
4. Use Eq. (9) to calculate $W_{pnl}$ (a branch cut tracking method is used to track full windings).

This is implemented in C++ for the WASP package, but the main routines can be accessed by a Python interface.

In addition, for comparison to existing calculations, we calculate the writhe as defined by:

13 $$\begin{aligned} Wr\equiv \frac{1}{4\pi }\int _\mathbf{x}\int _\mathbf{x}{} \mathbf{T}_{x}(s) \times \mathbf{T}_{x}(t)\cdot \frac{\mathbf{x}(s)-\mathbf{x}(t)}{{\Vert \mathbf{x}(s)-\mathbf{x}(t)\Vert }^{3}}\, ds\textit{ }dt. \end{aligned}$$

using the algorithm specified in^47^. This is the expression for writhing used in the closed ribbon Călugăreanu theorem (applied here to open curves). As discussed in the introduction, this measure has been used in open ribbon studies to characterise the writhing geometry of the ribbon’s axis and we present the results here for comparison. We do not present any results using closures. We highlight that whilst (13) is a valid quantity for open curves, it does not generally form an invariant sum with the twisting *Tw* and we lose the topological control associated with this invariance. In these plectoneme experiments (for example), this means the sum $W_p+Tw$ is exactly equal to the experimentally applied *Lk* whilst the sum $Wr +Tw$ is not. It was demonstrated in both^16^ and^26^ that there is always a closure for which the closed ribbon structure will have the exact same writhe value as $W_p$ (in fact it is the typical stadium closure). A numerical demonstration of this fact is also detailed in^26^. Further, it was shown that the closure can account for a significant proportion of the calculation, hence obscuring the interpretation. This last fact is an additional reason that the polar writhe local/non-local decomposition is a preferable measure for quantifying the ribbon’s evolving geometry in addition to the fact it forms part of an invariant sum for open ribbons whereas *Wr* does not.

## Results

### Undertwisted helices ($Lk = 40$)

**Figure 6:**
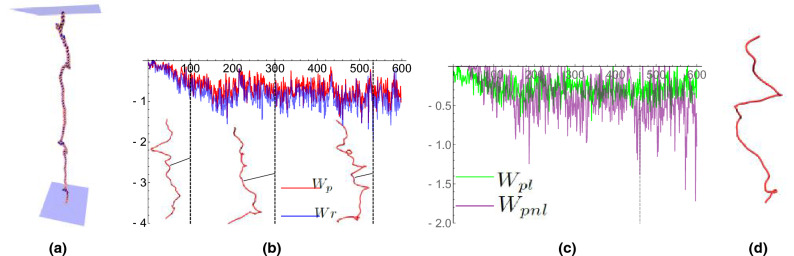
Results from simulations of an $Lk = 40$ DNA helix (undertwisted) placed under a 2pN extension force. Small kinked structures occasionally form along the length of the structure as shown in (**a**). Shown in (**b**) are writhe time series plots. The $W_p$ values are shown in red and the *Wr* values are shown in blue. The *Wr* values are consistently larger in magnitude but the variations in magnitude follow a very similar pattern. Example axis curves from the time series are shown; they correspond to the times marked by the dashed vertical lines. The curves’ width/height ratios are 0.3, chosen for clarity (0.1 would be the actual ratio). (**b**) A comparison of $W_{pl}$ and $W_{pnl}$ for the $W_p$ calculations shown in (**a**). There is a mixture of local $W_{pl}$ and non-local writhing values $W_{pnl}$ of roughly equal value. Some spikes in $W_{pnl}$ were found to arise from temporary loop formation. The dashed line indicates one such example whose curve is shown in (**d**). The spike in $W_{pnl}$ can be seen to correspond to the tight loop towards the bottom end of the curve.

Undertwisted helices exhibited relatively modest writhing dynamics throughout their trajectories. In contrast with torsionally relaxed helices, helical strain due to under-twisting results in the formation of small kink structures in the curve as indicated in Fig. 6a. We detail the results of two particularly pertinent cases here: a weak 2pN stretching force and a stronger 8pN force.

The 2pN results are shown in Fig. 6b. The values of both writhe measures *Wr* and $W_p$ settle at values of about $-1$, a twentieth of the applied (under) rotation. The $W_p$ value is consistently lower than the *Wr* value, although not by a substantial amount. As indicated in the figures, there is no plectoneme formation, however, partially looped sub-sections often form. The local/non-local decomposition of $W_p$ represents this mixture of helical (local) distortion and non-local coiling (see Fig. 6c). There are occasional spikes in the non-local writhing (relative to the local value). One such example is shown to arise from a tight loop formation as shown in Fig. 6d.

**Figure 7:**
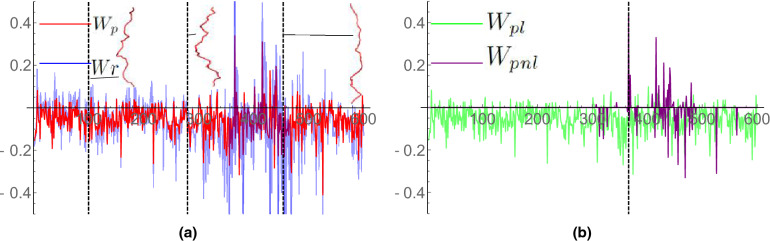
Writhe time series plots for an undertwisted DNA molecule ($Lk=40$) subjected to an 8pN pulling force. (**a**) A comparison of $W_p$ and *Wr* for the time series. The $W_p$ values are shown in red and the *Wr* values are show in blue. The *Wr* values are consistently larger in magnitude. Example axis curves from the time series are shown; they correspond to the times marked by the dashed vertical lines. The curves’ width/height ratios are 0.3, chosen for clarity (0.1 would be the actual ratio). (**b**) A comparison of $W_{pl}$ and $W_{pnl}$ for the $W_p$ calculations shown in (**a**). For the significant majority of curves, there is only local writhing.

The 8pN results are shown in Fig. 7a. The additional force further restricts the writhing of the curve and the magnitude of both quantities is typically bound between $[-0.5,0.5]$. The value of *Wr* is typically significantly larger than its $W_p$ counterpart. We verified that the ratio $|W_p-W_r|/(|W_p|+|W_r|)$ was larger than 0.5 for over a third of the calculations ($>50\%$ difference). In Fig. 7b we see that for vast the majority of the curve’s geometries there is only local writhing, a clear difference from the 2pN case which indicates that the force is acting to restrict loop formation. The non-local writhe values arose when small kinks in the structure developed, although again these are significantly restricted by comparison to the 2pN case. Intuitively speaking, in the context of this example, the local writhe is seen to reflect the vertical spring-like “bobbing” of the DNA in which the helix oscillates between stretching and compressing without any buckling/plectoneme formation occurring. The intermittency of the non-local writhe indicates the formation of buckling points that are not sustained, unlike the plectonemic formations in the following case.

### Overtwisted helices ($Lk = 80$)

**Figure 8:**
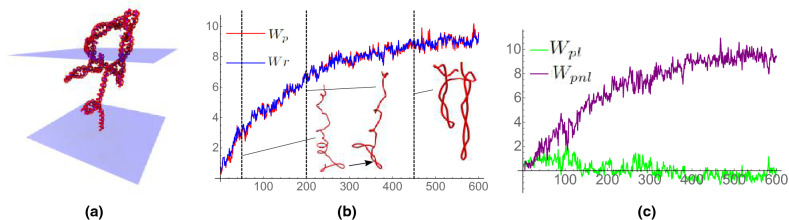
Results from simulations of an *Lk* = 80 DNA helix (over twisted) experiencing no extension force. In (**a**) we see the formation of significant supercoiling. In (**b**) we see that the development of this supercoiling is reflected by the steady increase in both writhing measures. Example axis curves from the time series are shown; they correspond to the times marked by the dashed vertical lines. The curves’ width/height ratios are 0.5, 0.4, 0.3 respectively and chosen for clarity (0.1 would be the actual ratio). (**c**) shows the local/non-local decomposition of $W_p$. Except in the initial stage, the non-local writhing is dominant and increases over time as the curve forms increasing numbers of plectoneme type loops.

Overtwisted helices exhibit dramatic writhing as a result of high torsional strain on the DNA helix. As a result, helices contract over time in the $\hat{z}$ direction by forming plectonemic structures (Fig. 8a). Time series plots of $W_{p}$ and $W_r$ are shown in Fig. 8b for a helix experiencing no extension force. There is a steady increase in writhing up to nearly 10 in both cases as the curve forms plectoneme structures. This is half the applied over-rotation which indicates that there has been a significant conversion of (over)twisting into writhing. We note that the two measures $W_p$ and *Wr* are nearly identical unlike in the under-twisted case. The reason for this is discussed in greater detail in the supplement. It suffices to state here that both assign the same value to the loops in the plectoneme structures which dominate the axis curve’s geometry.

The local/non-local decomposition is shown in Fig. 8c. Generally, except in the initial stage of simulation, there is far more non-local than local writhing, again due to the plectoneme formation. The consistent, gradual increase of the non-local writhe reflects the formation of plectonemes within the DNA helix. In contrast, the local writhe remains relatively constant, fluctuating only slightly due to the significant stress on the DNA helix causing any spring-like compression of the helix to be quickly converted into plectonemic buckling. In the first curve shown in Fig. 8b, sampled frame 50 has significant helical looping in its upper part. Near the lower end of the curve we see the initial formation of the first plectoneme, this contributes non-local writhing. As indicated in Fig. 8c, there is a reasonable balance of local and non-local writhing reflecting this two part geometry. The looped section has formed a plectoneme by timestep 200 as shown in Fig. 8b. By this stage, the non-local writhing is dominant (Fig. 8c). The final curve shown in Fig. 8b shows two plectonemes, the second developed from one of the small loops in the upper half of the curve at step 200.

### DNA minicircles

**Figure 9:**
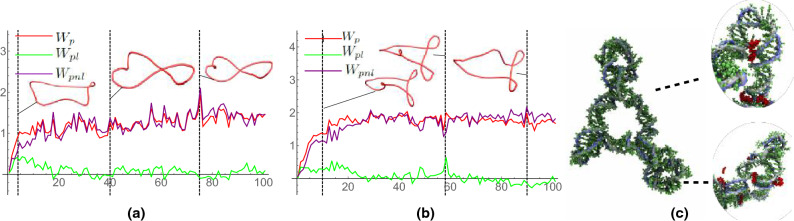
Polar writhe calculations for two DNA minicirlce cases. (**a**) indicates the writhing evolution of a 108 base pair $Lk = 14$ minicircle with a (GG)$_{27}$(GC)$_{27}$(GG)$_{27}$(GC)$_{27}$ sequence exposed to a 10 mM spermine cosolute concentration. (**b**) indicates the writhing evolution of a 108 base pair $Lk = 14$ minicircle with an (AA)$_{27}$(AT)$_{27}$(AA)$_{27}$(AT)$_{27}$ sequence exposed to a 10mM spermine cosolute concentration. (**c**) depicts spermine bridging in an (AA)$_{27}$(AT)$_{27}$(AA)$_{27}$(AT)$_{27}$ minicircle. Spermine molecules are shown in red. Spikes in $W_{pnl}$ in (**a**) indicate the formation of plectonemic loops within the DNA minicircle.

To further highlight the utility of $W_p$, we have included sample analyses of two DNA minicircles (Fig. 9). Both trajectories represent 108 base pair $Lk = 14$ minicircles exposed to a 10 mM spermine cosolute. The minicircle in Fig. 9a has the base pair sequence (GG)$_{27}$(GC)$_{27}$(GG)$_{27}$(GC)$_{27}$ and the minicircle in Fig. 9b has the sequence (AA)$_{27}$(AT)$_{27}$(AA)$_{27}$(AT)$_{27}$. In both trajectories, $W_{pnl}$ is observed to gradually increase throughout the beginning of the trajectory and then fluctuate around an average as the minicircle system reaches an equilibrium state. In these examples, the measurement of $W_{pnl}$ reflects the gradual formation of plectonemic loops/buckling points within the minicircle structures caused by spermine bridging. Spermine molecules adsorb to the backbone of the DNA minicircles early in the trajectories and gradually bridge together adjacent tracts of the DNA minicircles over time resulting in the formation of loops/buckling points within the minicircle structures as shown in Fig. 9c. The observed bridging dynamics are in accordance with results from previous studies as sequence dependent interactions have been observed between spermine molecules and AT/AA rich groups of linear DNA helices in which spermine molecules were observed to adsorb parallel to the DNA backbone and bridge together adjacent DNA helices^48^.

As shown in Fig. 9a, the axis of the GC/GG minicircle forms a figure 8 structure with a smaller loop occasionally forming at a consistent location on the curve. The $W_p$ value is generally dominated by the non-local component with variations being linked to the development (and reduction) of the smaller localised loop. In panel (b) it is shown that the AT/AA minicircle quickly forms two clear loops giving a $W_p$ value which oscillates around 2. Again there is a small localised loop which variably develops/recedes. At the point $t=49$ (marked with a dashed line) we see a relatively large spike in the local writhing corresponding to the loop localising and forming a tight local helical loop.

For both the AT/AA and GC/GG minicircles, the small localised loops that develop/recede within the minicircle structures are caused by spermine molecules that “jump” around the backbone of the DNA in various locations. These spermine molecules intermittently adsorb to the DNA backbone, bridging sections of the minicircle together which ultimately results in the formation of small loops that recede once the spermine molecules detach from the backbone and move to a new location. It is interesting to note that the non-local component of $W_p$ serves as a direct indicator of this phenomena, fluctuating directly in response to the aforementioned spermine/loop formation dynamics.

## Writhe star and winding classes

**Figure 10:**
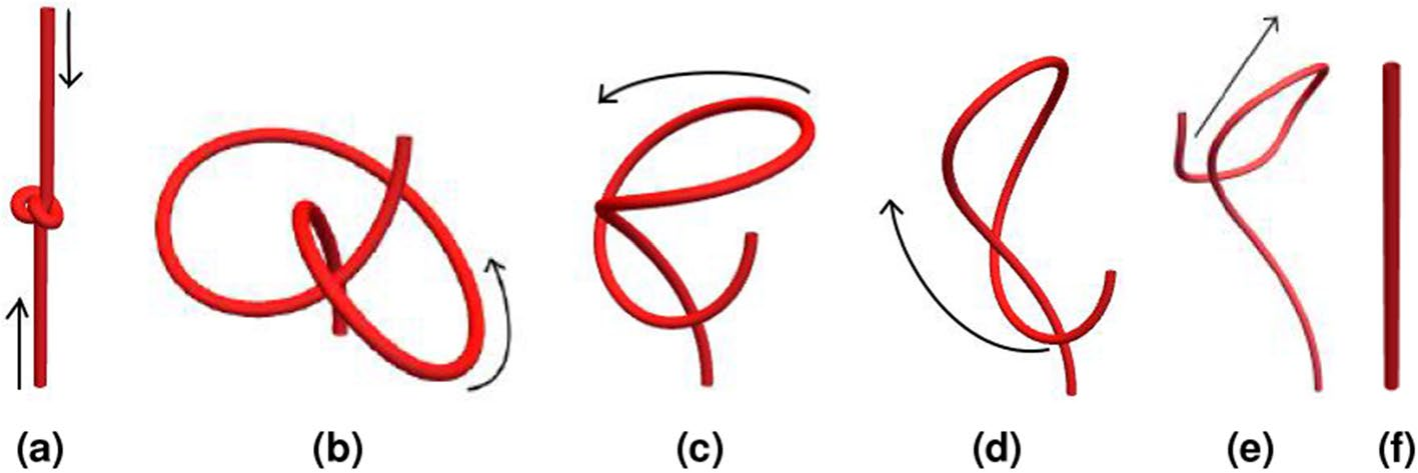
An “over-the-top” unknotting deformation. An initially tight knot shown in (**a**) is relaxed (**a**,**b**). A section of this knot is then allowed to loop over the top of one end of the curve (**b**–**d**). The curve is then pulled straight to an unknotted straight line (**d**–**f**).

A complication with open ribbons such as extended DNA structures is the possibility of (un)knotting and belt-trick type deformations such as those shown in Fig. 10^17^. These are classes of shape changes in which a section of the ribbon structure loops over one of its end points (as in Fig. 10b–d). In such cases, both *Lk* and $W_p$ can change by values of $\pm 2$^26^. Thus, when one prescribes a fixed value of *Lk*, it is only constrained up to an integer, but critically an integer change can only occur when an “over-the-top” deformation occurs. For many applications this will not be possible, for example, DNA structures in restricted environments and magnetic bead experiments where the bead itself is barrier. However, other potential applications of the writhing such as a measure of the development of tertiary structure in protein folding may need to consider such deformations. Prior and Neukirch^26^ considered how over-the-top deformations affect the polar writhe formulation; we briefly introduce that framework here through some examples of how this integer change can be tracked. This facility is built into the WASP package. *We should stress that if this over-the-top transition is prevented then one simply needs to use the* $W_p$ *calculations as described in the previous sections and what follows is unnecessary*.

### End angles and pulled-tight topology

**Figure 11:**
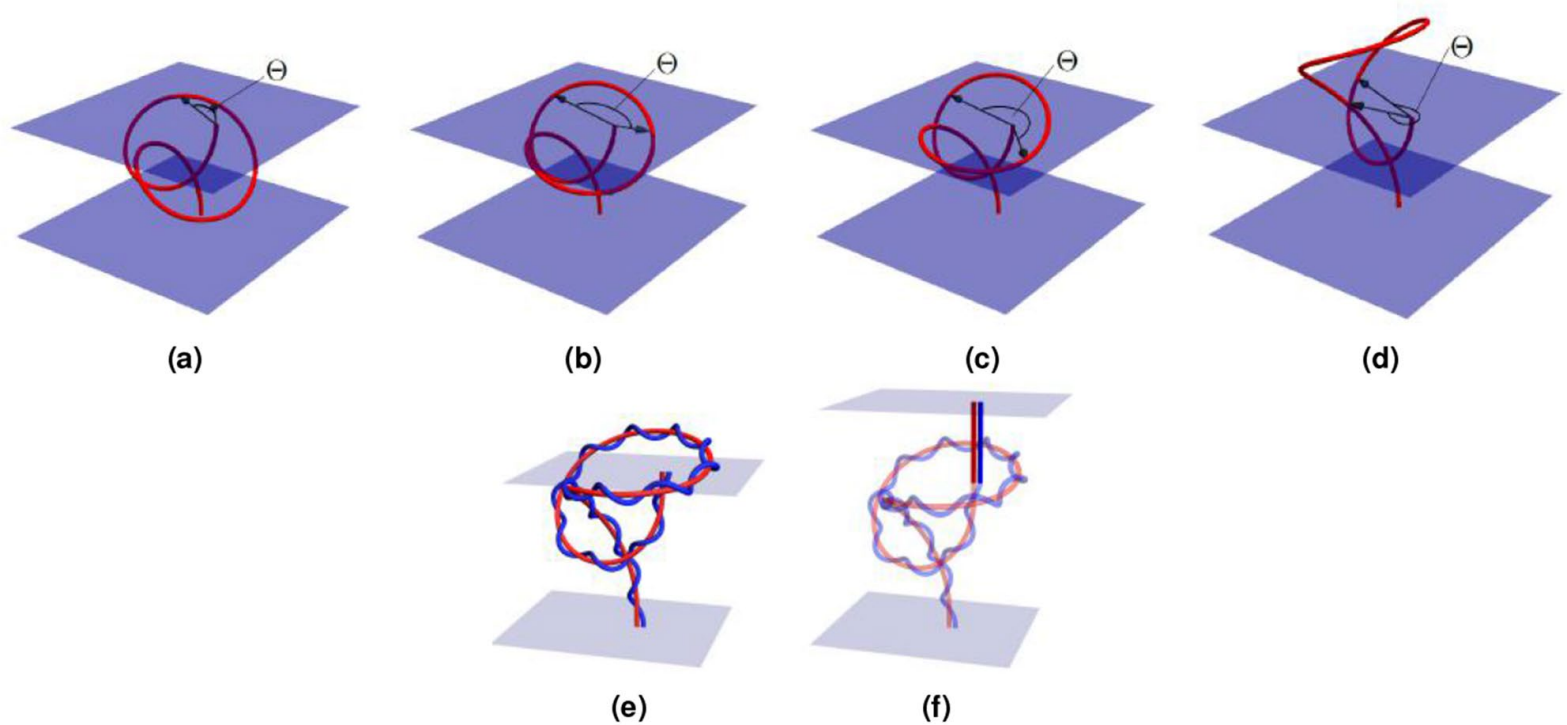
Illustrations of the loss in writhing which occurs to a section of the curve’s interior looping over one of its end points and the extension used to make this loss a discontinuous jump which tracks the pulled tight topology of the ribbon. (**a**) An interior section of the curve rises above one of the planes containing the curve’s endpoints. The angle $\Theta$ represents the difference of two contributions to the $W_{pnl}$ calculation which involves angles made with the curve’s end point. In (**b**,**c**) the curve section rises higher and the angle $\Theta$ increases. In (**d**) the angle is nearly $2\pi$ and the curve section has (just) passed directly over the top of the curve's end. (**e**) is a ribbon corresponding to one of the curves in Fig. 11; a section of the curve is in the plane above the ribbon’s end points. (**f**) The planar extension to the ribbon is shown. Now the whole ribbon structure is bounded between two planes. The extension is composed of planar curves with no twisting.

We can think of the two ends of an open ended ribbon as being bound between planes. If any section of curve passes through the end of these planes, it makes an angle with the end point as shown in Fig. 11a. This angle forms part of the non-local writhing calculation (Eq. (9)). As the curve loops over the top of the end point, this angle increases (see Fig. 11a–d). This change would affect both $W_p$ and *Lk*. If the curve then comes back down on the other side of the end point then this angle will have gone through a change of $2\pi$ and since this is counted twice it leads to a change in $\pm 2$ of both $W_p$ and $L_k$. Thus, overall the change of $\pm 2$ is detected.

However, this means that both $W_p$ and *Lk* would be changing continuously (although still such that $Lk- W_p=Tw$). This change does not happen if the curve is bound between the two end points where *Lk* is a fixed quantity. For magnetic field applications where the end planes represent the edge of the system, i.e. the sun’s surface^49,50^ or the edge of the measurement domain in a plasma experiment^51^, evaluating this angle is crucial. However, for plectoneme formation and protein topology type applications it is important that the section of curve leaving the bounding planes is included in the calculation. Prior and Neukirch^26^ derived a variant of the $Lk = W_p+Tw$ formula with quantities $W_p^*$ and $Lk^*$ which in effect ignore these end angles and just register a jump in both $W_p$ and *Lk*. More precisely, it was shown one could always extend the ribbon with straight sections (with no *Tw*) such that the ribbon remains bound between two planes. This adds no local writhing or winding and in effect remains a measure of the topology of the original unextended ribbon, except in one crucial aspect: when the curve/ribbon passes over its end point. In this case, the ribbon self intersects and there is a jump in $\pm 2$ in both *Lk* and $W_p$ and thus *Lk* is fixed up to integer multiples of 2. This has the following intuitive interpretation. If we imagine taking ribbon’s axis curve end points and pulling them directly apart, then a section of curve passing over the end point marks the point in its deformation where the curve would change its “pulled tight state”, i.e. Fig. 10a–f. For those readers familiar with the literature of artificial closures, there would be the same jump when the curve intersects its closure.

In summary, the sum

14 $$\begin{aligned} W_p^* +Tw, \end{aligned}$$

is invariant under all deformations which forbid curves passing over their end points and changes by values of $\pm 2$ when these over-the top deformations occur. This integer change can be used to indicate the ribbon’s pulled-tight topology (the shape obtained by pulling its ends directly apart) has changed.

### Examples

First we note that the parabola and looped example calculations shown in Figs. 2 and 4 would be identical, as would the undertwisted DNA results shown in Figs. 6 and 7.

#### Knot undoing

**Figure 12:**
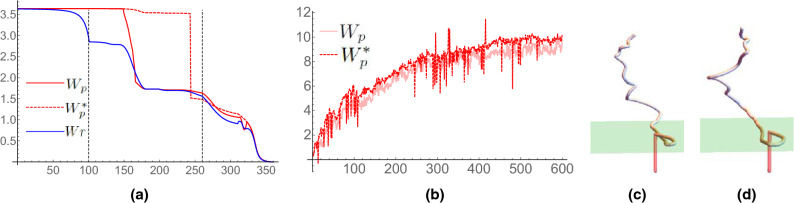
$W_p^*$ calculations. Panel shows (**a**) writhing values of the curves shown in Fig. 10. (**b**) A comparison of $W_p$ and $W_p^*$ for the overtwisted DNA simulations performed. (**c**,**d**) The detection of an over-the top deformation contributing one of the large jumps in the $W_p^*$ measure shown in (**b**). The end of the curve at neighboring timesteps and the end plane containing the point are shown in both (**c**,**d**). A red section of curve extending vertically downward from this point is shown (an extension used in the $W_p^*$ calculation in the WASP package). A section of the curve’s interior passes through this extension leading to a jump in the $W_p^*$ quantity.

The curves depicting the undoing of a knot in Fig. 10 are part of a continuous set of deformations. This deformation was discretized into 360 steps. The first 100 steps, Fig. 10a,b, are simple in that the shape of the knotted (trefoil) curve does not change but its size with respect to the straight end extensions is increased; in short, the shape of the curve is effectively unchanged. From curve 101 to 260, the knot is undone as a section of the curve loops over its top end point, Fig. 10b–d. Then, from step 261–360, the curve is pulled straight (Fig. 10d–f). The net effect is the undoing of a knot.

The values of $W_p$, $W_p^*$ and *Wr* (given by (13)) are shown in Fig. 12. For the first 100 steps, both $W_p$ and $W_p^*$ are identical and unchanged, correctly representing the fact the knot’s shape is essentially unchanged and the corresponding net winding would not change (assuming as always that the ends of the ribbon are prevented from untwisting). The *Wr* calculated via the Gaussian integral (Eq. (13)), however, does change. The reasons for this change are somewhat technical and are discussed in the supplement; it suffices to note here that it is not characterizing the knots unchanging shape and the sum $W_r+Tw$ would not be a fixed quantity.

The set of writhe values for configurations 101–260 (the over-the-top unlooping of the knot) highlight the difference between $W_p$ and $W_p^*$. For the first part of this deformation they are identical until the looped section passes above the end point’s plane (as shown in Fig. 11). Then $W_p$ begins to measure a changing value due to the end angle measures between this loop and the end point. $W_p^*$ does not account for this change. Near the end of this un-looping, the curve passes directly over the top of its end point and we see a jump of $-2$ in $W_p^*$ where the measure has classed the curve as jumping into a different pulled tight topological class. In the final set of deformations 260–360, the curve is pulled straight; here all three measurements are in rough agreement that there is a steady decrease in writhe. Note, however, that $W_p^*$ measure registers a relatively smooth change.

### Overtwisted DNA reconsidered

The discontinuous jump in the $W_p^*$ measure can lead to jumpy time series **if** a section of the curve is continually deforming in the region directly above one of the curve’s end points. It happens that this is exactly what is occurring in the overtwisted plectoneme formation simulation in the previous section. The comparison of $W_p$ and $W_p^*$ is shown in Fig. 12b. The $W_p^*$ measure has some significant discontinuities; each one was analyzed and in each case it was found to result from a section of the curve passing over the bottom end of the curve. An example is shown in Fig. 12c,d.

A second observation is that $W_p^*$ is consistently a small amount larger than $W_p$. This is due to a difference between $W_{pnl}$ and $W_{pnl}^*$ ($W_{pl}$ is always the same for both), specifically the ignoring of the end angle depicted in Fig. 11. It is interesting that the molecule appears to deform such that it partly performs the over-the-top loop, a potential means to shed topology, but never completes this deformation, instead forming a second plectoneme loop.

A final mathematical point is that if one only calculated $W_p^*$ for the given curve set, then, without looking at the individual curves themselves, it is hard to tell if the jump occurs due to self crossing or over the end deformations. The $W_p$ calculations, which have no changes on the order of 2 indicate it is not self-crossing but over the top deformation instead. Thus, one can make this distinction solely from the numerical calculations (rather than visulising the curve set). Of course we should have expected this for the DNA model used as repulsive forces prevent self-crossings, but in other scenarios the combination of information provided by both the $W_p$ and $W_p^*$ quantities could be of significant utility.

The previous two examples highlight aspects of the $W_p^*$ measure which the user should consider when deciding if it is appropriate for their application. The $W_p^*$ measure was explicitly designed for elastic rod models in which both knotting and belt-trick style deformations clearly occurred: the end state had unambiguously changed. The pulled tight definition is designed to capture this change. The plectoneme simulation cautions that this change may not always be meaningful. In addition, it is also possible that the net change in $W_p^*$ in some knotting deformation might add up to zero (an equal number of positive and negative jumps) even though the essential knotting entanglement might have changed. The WASP package gives the user the option to calculate both $W_p$ and $W_p^*$ and we believe these examples will provide a guide as to how to interpret these measures.

## Conclusions

Writhe is a fundamental measurement for characterizing the topology of ribbon structures and is extensively utilized in multiple fields ranging from biophysics to solar physics to aneurysm detection (to name just a few)^30–32,52,53^. However, the most commonly utilized formalism for writhe (as derived by Călugăreanu ) is only applicable to the subset of problems in which the system under study is a closed loop. Although this limitation has been overcome through the development of polar writhe, it is largely underutilized in the biophysical community. Here, we have sought to rectify that problem by demonstrating the utility of polar writhe in DNA plectoneme and DNA minicircle calculations. Our results show that polar writhe is not only applicable to the analysis of open curves such as those formed by extended DNA structures but that it can be decomposed into local and nonlocal contributions that can provide additional information about DNA topology that cannot be obtained from the Călugăreanu formalism. In particular, we demonstrate how the non-local component of the polar writhe can be used as a potential indicator of plectoneme formation in both linear and circular DNA structures which we believe will be of particular value to the host of studies performed on effects of plectoneme formation in DNA and braided polymers^54,55^. To aid in the adoption of polar writhe, we have developed a software package, WASP, that can be used to analyze molecular dynamics simulations of DNA. WASP is an open source software tool that is built to analyze trajectories from popular simulation packages. It is our hope that WASP will be utilized to rigorously analyze the growing field of DNA simulations in which changes of writhe are linked to biophysical phenomena (Fig. S1).

## Supplementary information

Supplementary Information.

## Data Availability

The source code for WASP is located in the following repository: https://github.com/WereszczynskiGroup/WASP
