# Supplementary material for: WASP: a software package for correctly characterizing the topological development of ribbon structures
Source: Sci Rep. 2021 Jan 15;11:1527. doi: 10.1038/s41598-020-80851-8 (PMC7811023; doi:10.1038/s41598-020-80851-8)
Supplement: Supplementary file 1 — Supplementary Information. [file 41598_2020_80851_MOESM1_ESM.pdf]

WASP: A software package for correctly characterizing the topological development of ribbon structures: Supplementary material

Zachary Sierzega[1,2], Jeff Wereszczynski[1,*], and Chris Prior[3]

[1]Illinois Institute of Technology, Department of Physics and The Center for Molecular Study of Condensed Soft Matter, Chicago, IL, 60616, United States
[2]Current Address: University of Colorado, Boulder, Department of Physics, Boulder, CO, 80309, United States
[3]Durham University, Mathematical Sciences, Durham, DH1 3LE, United Kingdom

ABSTRACT

Supplementary information to: WASP: A software package for correctly characterizing the topological development of ribbon structures. Here the polar writhe measure is related to existing writhe measures used in the literature.

Ribbons and the open and closed Călugăreanu formulae

In this section we detail how the polar writhe formulation relates to the closed invariant relationship first introduced by Călugăreanu[1]. We further relate it to the Fuller formulae which have been used in DNA supercoiling and elastic rod applications[2–7].

We begin by considering a twice differentiable space-curve $\mathbf{x}(s) \in \mathbb{R}^3$, $s \in [0,L]$, which is closed ($\mathbf{x}(0) = \mathbf{x}(L)$) and parameterized by its arclength s. Its unit tangent vector is denoted $\mathbf{T}$. A ribbon structure is constructed using a vector field $\mathbf{V}$ normal to $\mathbf{x}$ ($\mathbf{V}\cdot\mathbf{T} = 0, \forall s$). This field may rotate around the curve $\mathbf{x}$ and defines a second curve $\mathbf{y}$, the ribbon's edge

$$\mathbf{y}(s) = \mathbf{x}(s) + \varepsilon\mathbf{V}(s), \tag{1}$$

as illustrated in Figure 1(a). It is usually assumed ε, the ribbon's width, is significantly smaller than the length of the curve $\varepsilon \ll L$. At first we also assume the ribbon is closed ($\mathbf{x}(0) = \mathbf{x}(L)$ and $\mathbf{y}(0) = \mathbf{y}(L)$). Note that for DNA molecules we are typically assuming the axis curve $\mathbf{x}(s)$ is the supercoiling axis of the structure, not the helical axis, *i.e.* a circle in a relaxed mini ring or a straight line for linear DNA (see Figure 1(b)).

The link-twist-writhe theorem for closed ribbon's first introduced by Călugăreanu relates three quantities

$$Lk(\mathbf{x},\mathbf{y}) = Tw(\mathbf{x},\mathbf{V}) + Wr(\mathbf{x}), \tag{2}$$

the linking number Lk of the ribbon's edges, the writhe Wr of its axis, and the twist Tw of the vector field $\mathbf{V}$. We now formally introduce these quantities.

Linking Number, writhe and twist of closed ribbons

The linking number Lk of a ribbon in $\mathbb{R}^3$ can be defined by the Gaussian integral:

$$Lk(\mathbf{x},\mathbf{y}) = \frac{1}{4\pi}\oint_x\oint_y \mathbf{T}_\mathbf{x}(s)\times\mathbf{T}_\mathbf{y}(t)\cdot\frac{\mathbf{x}(s)-\mathbf{y}(t)}{\|\mathbf{x}(s)-\mathbf{y}(t)\|^3}\,\mathrm{d}s\,\mathrm{d}t, \tag{3}$$

where $\mathbf{T}_\mathbf{x}$ and $\mathbf{T}_\mathbf{y}$ are the unit tangent vectors of $\mathbf{x}(s)$ and $\mathbf{y}(t)$, respectively. Lk is an integer valued quantity and most importantly a topological invariant, meaning that any continuous invertible deformation of the ribbon does not change Lk. Stretching the ribbon is an example of a such a deformation; cutting the ribbon is not. If the curves $\mathbf{x}$ and $\mathbf{y}$ are allowed to pass through itself the linking number changes by ± 1[8]. The crossing of the ribbon with itself is always means two crossings of $\mathbf{x}$ and $\mathbf{y}$, so there will be a change ± 2 in Lk if a ribbon crosses itself.

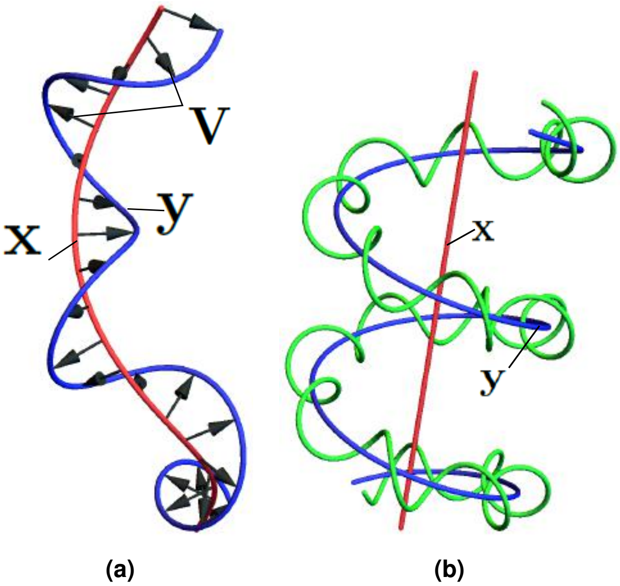

Figure 1. Ribbon structures. Panel (a): a ribbon is composed of an axis curve $\mathbf{x}(s)$ and a second curve $\mathbf{y}(s)$ constructed from the axis curve and a vector field $\varepsilon\vec{V}(s)$. In panel (b) we see a ribbon structure as applied to an idealized DNA coiled-coil geometry. The axis $\mathbf{x}$ is the straight line at the centre of the coil and the second curve $\mathbf{y}$ is the helical curve at the centre of the DNA's coiled-coil geometry. The green curve might represent one strand of the DNA polynucleotide chain.

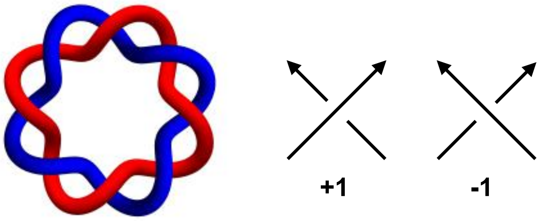

Figure 2. A (2,8) torus link with $Lk = Tw = 4$ and $Wr = 0$. Lk can be calculated for the planar in-page projection using $Lk = \frac{\sum C_+ + C_-}{2}$ following the sign convention as pictured.

Linking, the Gauss map and points of view

The double integral (3) represents the degree of the Gauss map:

$$\mathscr{C}(s,t) = \frac{\mathbf{y}(s) - \mathbf{x}(t)}{\|\mathbf{y}(s) - \mathbf{x}(t)\|}, \tag{4}$$

applied to all s and t for the two curves. The map takes pairs of points $(\mathbf{x}(s), \mathbf{y}(t))$ to points on the unit sphere $\mathbb{S}^2$, that is to say it defines directions between points (*e.g.*[9]). Equation (3) is an application of the Kronecker formula (*e.g.* Chp 8.3[10]) and each point on the unit sphere is assigned a positive or negative value by the sign of the integrand. A visual interpretation of this calculation is instructive: consider a (2,8) torus link as shown in Fig. 2. An observer may project the torus link directly into the page along some fixed direction $\mathbf{n}$ and calculate Lk for the given projection by adding the signed number of instances in which the red and blue edge curves cross, following the under/over-crossing sign convention indicated in figure 2, then dividing the sum by two. The crossings will coincide with pairs of points (s,t) at which $\mathscr{C}(s,s') = \mathbf{n}$. The sign rule gives the sign of the scalar triple product $\mathbf{T_x}(s) \times \mathbf{T_y}(t) \cdot (\mathbf{x}(s) - \mathbf{y}(t))$. It can be shown that, whatever the direction of projection $\mathbf{n}$, the value of Lk calculated by this method will be the same[10]. The integral (3) can thus be seen as the average value of this projection method over all possible choices of direction $\mathbf{n}$ (all points on the unit sphere) and is thus is a highly degenerate calculation (in the sense that this averaging is unnecessary and one can calculate the integer quantity Lk from a single projection). Another

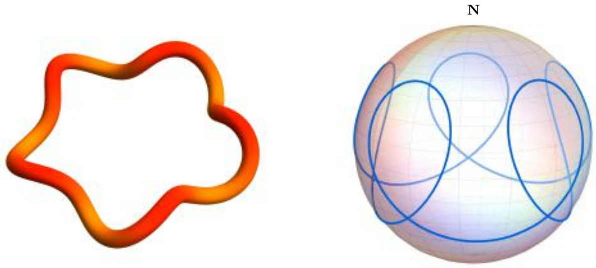

Figure 3. A (2,10) torus knot (Left) and its unit tangent curve $\mathbf{T}$ (Right); this is one edge of an area on the surface of the unit sphere which represents the calculation (7). Changes in shape of the curve on the left would lead to changes in this unit tangent curve, thus changing the area it bounds. This change represents the changing value of Wr under deformation.

way of stating this is that the Gauss integral 3 is not the fundamental definition of the linking number but one possible method of calculating it. An example alternative method was proposed by Banchoff calculating this integer for polygonal ribbons[11]).

The net winding

The net winding of a pair of curves $\mathbf{x}$ and $\mathbf{y}$ is defined as follows. We split $\mathbf{x}$ into n sections by its turning points along $\hat{z}$ and similarly split $\mathbf{y}$ into m sections by its turning points (as discussed in section 2 of the main document). For each pair $(\mathbf{x}_i, \mathbf{y}_j)$ we can calculate the mutual winding of the angle Θ_{ij} made by the vector $\mathbf{y}_j(z) - \mathbf{x}_i(z)$ **if** they share a range of z values $z \in [z_{ij}^{min}, z_{ij}^{max}]$. Then the net winding is the sum of all these contributions:

$$L(\mathbf{x},\mathbf{y}) = \sum_{i=1}^{n}\sum_{j=1}^{m} \frac{\sigma_i \sigma_i}{2\pi} \int_{z_{ij}^{\min}}^{z_{ij}^{\max}} \frac{\mathrm{d}}{\mathrm{d}z}\Theta(\mathbf{x},\mathbf{y},z)\,\mathrm{d}z. \tag{5}$$

For closed ribbons this formula can be shown equal the integral (3) ($L = L_k$), however, it is not the same calculation. We can define the map:

$$\mathscr{C}_z(z) = \frac{\mathbf{y}(z) - \mathbf{x}(z)}{\|\mathbf{y}(z) - \mathbf{x}(z)\|}, \tag{6}$$

which is very similar to the Gauss map $\mathscr{C}(s,t)$, except it restricts to points on the curve which have the **same** z coordinate. It was shown in[12] that (5) is equal to the average of the linking calculated by projection for all directions $\mathbf{n}$ which lie in the plane *i.e.* $\mathbf{n} = (\cos(\Theta), \sin(\Theta), 0), \forall \Theta \in [0, 2\pi]$. When $\mathscr{C}_z = \mathbf{n}$ one would see a crossing of the curves. This is obviously a far less degenerate calculation than (3), but given *Lk* can be calculated from **one** projection, we might ask why this averaging is necessary? That is to say why is the net-winding an interesting quantity in its own right? The answer is two-fold. First, for open ribbons, the linking calculated by a single projection is **not** invariant to all motions which vanish at the ribbon's end (we will deal with this matter shortly). Second, using (5) yields a sensible writhing definition which is equal to *Lk*-*Tw*, as we now discuss.

The Writhe Wr

The writhe Wr can also be defined as a Gaussian type integral, in effect the self-linking of the curve $\mathbf{x}(s)$:

$$Wr \equiv \frac{1}{4\pi}\oint_x \oint_x \mathbf{T}_{\mathbf{x}}(s) \times \mathbf{T}_{\mathbf{x}}(t) \cdot \frac{\mathbf{x}(s) - \mathbf{x}(t)}{\|\mathbf{x}(s) - \mathbf{x}(t)\|^3}\,\mathrm{d}s\,\mathrm{d}t. \tag{7}$$

Unlike the linking number, writhe is dependent on only the axis curve of the ribbon and is not a topological invariant[9,12]. Similar to the linking number, (7) can be interpreted as the area on the unit sphere covered by the map

$$\mathscr{C}_w(s,t) = \frac{\mathbf{x}(s) - \mathbf{x}(t)}{\|\mathbf{x}(s) - \mathbf{x}(t)\|}, \tag{8}$$

with the area element positive or negative by the sign of the integrand $\mathbf{T}_x(s) \times \mathbf{T}_x(t) \cdot (\mathbf{x}(s) - \mathbf{x}(t))$. However, this area is not a degree (it is not an integer), and not a topological invariant[9,12]. In the limit $t \to s$ from below, $\mathscr{C}_w$ tends to the unit tangent vector $\mathbf{T}(s)$ and in the limit $t \to s$ from above it tends to $-\mathbf{T}(s)$. By contrast, the linking map $\mathscr{C}$ has the same limit $t \to s$ from above or below and is periodic. So the area covered by the linking integral, equation (3), will be a closed surface (and hence an

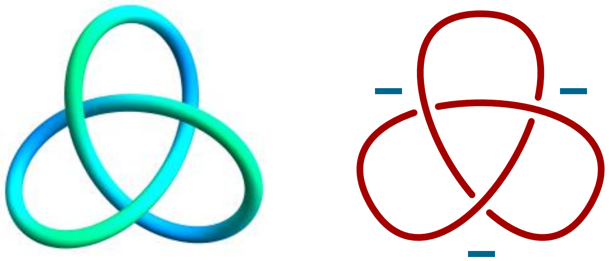

Figure 4. Left-handed trefoil knot with $wr = -3$ and $Wr = -3.2$. The planar writhe wr can be calculated using the sign convention in Fig. 2.

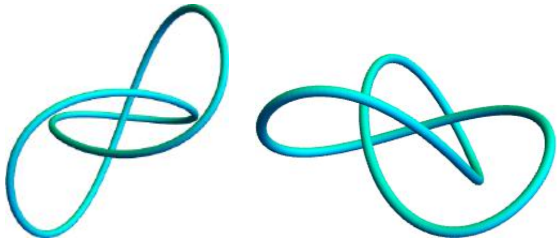

Figure 5. Two alternative projections/viewpoints of the trefoil knot shown in Figure 4. The left view point would lead to a projection with $wr = -3$ and the right a projection for which $wr = -4$.

integer number of signed coverings of the unit sphere), whilst the area covered the writhe integral (equation 7) has a boundary which is the union the unit tangent curves $\mathbf{T}(s)$ and $-\mathbf{T}(s)$ mapped onto the sphere[9] (an example is shown in Figure 3). The Wr changes continually under continuous (invertible) deformations of the ribbon (as the tangent curve $\mathbf{T}$ changes), and when x crosses itself Wr changes by a value of ± 2.

Similar to Lk, Wr can be interpreted in terms of planar projections. For example, a left-handed trefoil knot and a possible planar projection are shown in Figure 2. Walking along the projected curve and counting the signed self crossings using the same convention as in figure 2 gives a planar (projected) writhe of $wr = -3$. However, in contrast to the linking, alternative projections **can** lead to changes in the planar writhe as indicated in Figure 5. The writhe Wr is an average over ***all*** possible projections (all planar wr) and in the trefoil example $Wr = -3.2$, indicating the bulk of projections have $wr = -3$ for the given trefoil knot. Changing the shape of the trefoil will alter the percentage of $wr = -3$ and $wr = -4$ cases and hence change the average. We highlight that it is the averaged quantity (7) which is required for (2) to hold.

Polar writhe

The polar writhe introduced in section 2 of the main document yields exactly the same value as Wr evaluated by (7), as demonstrated in[12]. Similar to the net winding, the polar writhe can be shown to be equal to the planar writhe Wr averaged over all projection directions $\mathbf{n}$ which lie in the plane ($\mathbf{n} = (\cos(\Theta), \sin(\Theta), 0), \forall \Theta \in [0, 2\pi]$). As indicated in Figure 4, the projected writhe wr does not equal Wr so the sum $wr + Tw$ would not be invariant to non self intersecting deformations of the ribbon. As demonstrated in[12], it is necessary to average over all planar directions in order to obtain the quantity $W_p = Wr$ which makes equation 2 hold. That is to say the polar writhe is the most parsimonious method for calculating Wr (for closed curves). In[13] the polar writhe code underlying the WASP package was compared to various existing methods for calculating Wr[14,15]. It was found that the polar writhe method was the consistently the fastest, scaling linearly in the number of points of the discrete curve representing the ribbon's axis.

The Twist Tw

Finally, the twist of a ribbon is equal to the total rotation of the generating vector field $\mathbf{V}$ around the tangent direction of the axis curve $x(s)$:

$$Tw \equiv \frac{1}{2\pi} \oint_{\mathbf{x}} \mathbf{T} \cdot \mathbf{V} \times \frac{\mathrm{d}\mathbf{V}}{\mathrm{d}s} \, \mathrm{d}s. \tag{9}$$

It changes continually under continuous deformations of the ribbon, even those which intersect[12].

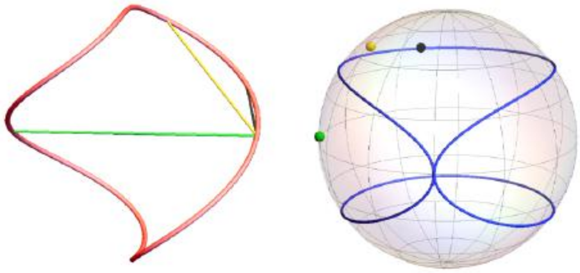

Figure 6. Various contributions to the Wr calculation which can be classed as local or non-local. On the left a curve and a subset of its chords $\mathscr{C}_w(s,t)$ are shown. The parameters s and t are chosen so these chords vary from clearly non-local (green) to clearly local (black), and somewhere in between (yellow). On the right we see the unit tangent tangent curve $\mathbf{T}$ of the curve on the left, plotted on the unit sphere. The points corresponding to the chords shown on the left indicate their relative locality by their proximity to the unit tangent curve (the nearer to this curve the more we might say they are local)

Again considering the torus knot example of Fig. 2: because the axis curve for this torus knot is easily determined to be a planar circle, it can be seen that $Wr = 0$ for this given geometry and thus $Lk = Tw$ by (2).

If the vector $\mathbf{V}$ happens to be the normal vector of $\mathbf{x}$, which is the unit vector pointing along the direction of the derivative of the Ribbon's tangent vector $\mathrm{d}\mathbf{T}/\mathrm{d}s$, then the twist is called the torsion and the Călugăreanu theorem 2 is referred to as the self linking formula (see *e.g.*[16]). This name derives from the fact that the ribbon's geometry is entirely determined by the shape of the axis $\mathbf{x}$. The theorem 2 is far more general as it allows for a genuinely independent degree of freedom of rotation of the vector $\mathbf{V}$[13].

Local and non local writhing and the Fuller formulae

Local and non-local crossings

One might categorise contributions to the Wr calculation (7) from chords $C_w(s,t)$ for which $|s-t| > \varepsilon$, for some small ε, as non-local. However, as indicated in Fig 6, there are contributions which are in some sense very clearly non-local which can be used to measure the mutual entanglement of clearly distinct sections of the curve, whilst there are others which might be classified as non-local ($|s-t| > \varepsilon$) but which are determined by relatively small subsections of the curve. Put simply, a sensible value of ε would be highly curve dependent and this attempt to categorise contributions as local and non-local is not precise. To the best of our knowledge the only precise attempts to do so are based on the Fuller fomrulae introduced in[2]. As we have demonstrated in section 2 of the main document, there is a natural local/non-local separation in the polar writhe calculation which **can** provide significant extra geometrical information. For the sake of completeness we now detail (briefly) how the popular Fuller writhe expressions relate to this decomposition.

The Fuller Formulae

As mentioned above, the unit tangent curve $\mathbf{T}$ of the ribbon's axis and its negative image $-\mathbf{T}$ are the boundaries of the area bound on the unit sphere which represents the calculation (7). Fuller[2] proposed that the total signed area $\mathscr{A}$ closed by the curve $\mathbf{T}$ on the unit sphere characterises Wr up to an integer multiple of 2:

$$W_F = \frac{\mathscr{A}}{2\pi} - 1 = Wr \quad mod\, 2. \tag{10}$$

This proposition was proven in[17]. While (10) is determined entirely by the local differential geometry of $\mathbf{x}(s)$ through the curve $\mathbf{T}$, it partly represents the non local contributions, as we will shortly discuss. W_F does not fully account for (net) signed full coverings of the sphere involved in the calculation of equation (7)[12]. For example, the Fuller formula correctly evaluates the Wr of the curve shown in Figure 6, but if we apply an extra half rotation (right handed) to the curve, as indicated in Fig 7, the Fuller formula misses the correct writhe value by 1. As a result, despite the existence of $\mathscr{O}(N)$ methods for computing writhe, W_F cannot be treated as equivalent to Wr as demonstrated in[18].

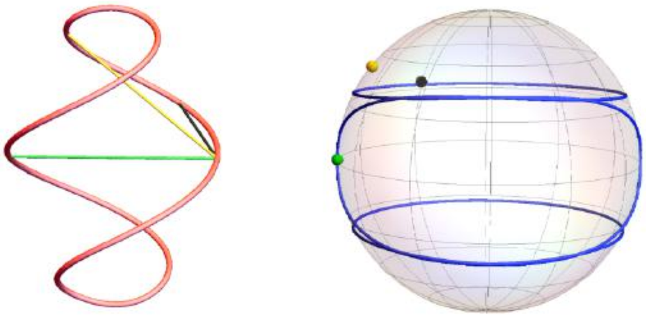

Figure 7. An illustration of a curve for which $W_F \neq Wr$. The curve (left) is obtained form the curve in Figure 6 by applying and extra half twist at its ends. A set of chords with the same (s,t) as those in Figure 6 are shown. We note the most clearly non-local chord lies on the unit tangent curve $\mathbf{T}$ (right). This is only possible because there is a full net rotation of the curve which leads to an extra full covering of the unit sphere in the calculation of Wr. Such contributions are missed in the evaluation of W_F (it is part of an area not bounded by $\mathbf{T}$).

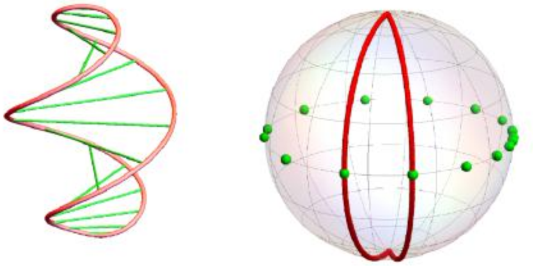

Figure 8. A visual interpretation of W_{pnl}. The sets of chords $\mathscr{C}_w(s,t)$ for which the z component of $x(s)$ and $x(t)$ are equal are used to track full rotations of distinct sections of the curve separated by "turning points" for which $\mathrm{d}s/\mathrm{d}z = 0$. Such chords correspond to points on the equator of the unit sphere (right) and can be characterised by an angle coordinate Θ (right). The change in angle $\Delta\Theta$ between two of the pictured chords can be assigned a segmental area bound by two geodesics joining the respective angles to the poles (right). If the chord undergoes a full rotation then, as indicated, the the total area will include one full covering of the sphere.

The polar writhe and unit sphere areas

It was shown in[12] that the non local polar writhe of a closed curve $\mathbf{x}$ with n sections $\mathbf{x}_j$ split by its turning points can be calculated as follows:

$$W_{nl}(x) = \frac{1}{2\pi}\left[\sum_{k=1}^{n-1} 2\,\sigma_i\sigma_{i+1}\,\phi_k + \sum_{\substack{i=1\\ i\neq j}}^{n}\sum_{j=1}^{n}\sigma_i\sigma_j n_{ij}\right] - 1, \tag{11}$$

where $\phi_k \in [0, 2\pi)$ is the angle made by $\mathbf{T}$ with the x-axis at a turning point. These contributions arise from the integrals

$$\int_{z_{ii+1}^{min}}^{z_{ii+1}^{max}} \frac{\mathrm{d}\Theta_{i(i+1)}}{\mathrm{d}z}\mathrm{d}z, \tag{12}$$

which connect at the mutually shared turning point of the sections $\mathbf{x}_i$ and $\mathbf{x}_{i+1}$. The factor of -1 accounts for the periodicity of the closed curve as there is a contribution from the pair $\mathbf{x}_1$ and $\mathbf{x}_n$ whose mutual angle is $\pi+\phi_1$. Further it was shown (in Theorem 4 of[12]) that the $\mathscr{A}$ in the Fuller formula (10) is given by

$$\mathscr{A} = \frac{1}{2\pi}\sum_{k=1}^{n-1} 2\,\sigma_i\sigma_{i+1}\,\phi_k + W_{pl}, \quad (\text{mod } 2) \tag{13}$$

(for closed curves). Thus we see that the quantity represented by the Fuller writhe formula is the local polar writhe plus a term which can be calculated from the local geometry of the curve, but actually represents a non local quantity, the turning point tangent angles ϕ_k. For example, in the curve shown in Figure 6, the difference in the tangent angles of the upper and lower apexes of the curve give exactly W_{pnl} (not W_{pl}). This is illustrated clearly in Figure 8 where a set of non local chords joining sections of the curve of equal z height rotate in z. The calculation of W_{pnl} for these rotating chords is equal to the area swept out by a pair geodesics joining the poles on the unit sphere bounding the areas between the angles made by these chords. The top and bottom angles are the limiting angles ϕ_k; their difference gives W_{pnl} up to an integer and so characterises part of the non local writhing.

The Fuller ΔWr formula

Fuller proposed a second formula for the difference in writhing between two closed curves $\mathbf{x}$ and $\mathbf{x}'$. If we can deform $\mathbf{x}$ into $\mathbf{x}'$ smoothly such that for all the intervening curves their tangents never oppose $\mathbf{T}\cdot\mathbf{T}' \neq -1\,\forall s$ then:

$$Wr(\mathbf{x}) - Wr(\mathbf{x}') = \oint \frac{\mathbf{T}'\times\mathbf{T}}{1+\mathbf{T}\cdot\mathbf{T}'}\cdot\left(\frac{\mathrm{d}\mathbf{T}}{\mathrm{d}s} + \frac{\mathrm{d}\mathbf{T}'}{\mathrm{d}s}\right)\mathrm{d}s. \tag{14}$$

A rigorous proof for this was given in[17]. The so called non opposition condition $\mathbf{T}\cdot\mathbf{T}' \neq -1$ means the two curves cannot differ by a looped section (*i.e.* plectonemes). Since $W_p = Wr$, (14) can also be applied to the polar writhe. It was shown in[12] that the difference $W_p(\mathbf{x}) - W_p(\mathbf{x}')$ can be characterised by the difference in local writhing W_{pl} and the tangent angles ϕ_k. Specifically, if we write

$$W_p^{\dagger}(\mathbf{x}) = W_{pl}(\mathbf{x}) + \frac{1}{2\pi}\sum_{k=1}^{n-1} 2\,\sigma_i\sigma_{i+1}\,\phi_k. \tag{15}$$

Then

$$\begin{aligned} W_p(\mathbf{x}) - W_p(\mathbf{x}') &= W_p^{\dagger}(\mathbf{x}) - W_p^{\dagger}(\mathbf{x}') \\ &= \oint \frac{\mathbf{T}'\times\mathbf{T}}{1+\mathbf{T}\cdot\mathbf{T}'}\cdot\left(\frac{\mathrm{d}\mathbf{T}}{\mathrm{d}s} + \frac{\mathrm{d}\mathbf{T}'}{\mathrm{d}s}\right)\mathrm{d}s, \end{aligned}$$

(if the non-opposition condition holds). So we see the second Fuller formula conveys the same mixture of local and non-local information as the area formula, but **not** the full local non-local decomposition of the polar writhe.

Link twist and writhe of open ribbons

In this section we consider the topology of ribbons bound between two planes whose end pairs $(\mathbf{x}(0), \mathbf{y}(0))$ and $(\mathbf{x}(L), \mathbf{y}(L))$ lie in the bounding planes (as in Figure 9). If its ends are prevented from rotating and it deforms without crossing itself we should expect the mutual linkage of the two ends of the ribbon to be conserved. As discussed in the introduction and proven in[12], the net winding given by (5) (which is equally applicable to open ribbons) **is** invariant if the ends of the ribbon are prevented from rotating. Further it is shown that

$$L(\mathbf{x},\mathbf{y}) = W_p(\mathbf{x}) + Tw(\mathbf{x},\mathbf{V}). \tag{16}$$

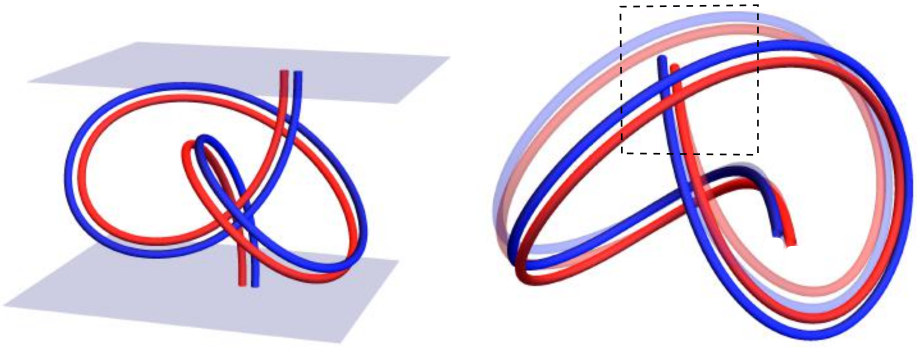

(a) **(b)**

Figure 9. Open ribbons. Panel (a): an open ended ribbon whose end points stay fixed but whose interior might deforms smoothly. Panel (b): The ribbon is observed from a particular point of view included in the evaluation of Lk but not the net-winding L. One can calculate the planar linking of this projection by assigning an orientation to the curves using the crossing rules indicated in Figure 2. The value changes in the subsequent deformation of the interior of the ribbon (shown opaquely) as we see a set of crossings is lost in the opaque figure as a section of the interior passes over the ribbon's end.

Gaussian linking/writhing?

Another suggestion in the literature has been to use Wr, defined by (7), which can be applied to open curves, as a means of characterising the ribbon's topological development. This is why calculations of this quantity were compared to polar writhe calculations on the DNA models in the main paper. There are two significant drawbacks to this approach. First, there is no known proof that the sum $Wr(\mathbf{x}) + Tw(\mathbf{x}, \mathbf{V})$ is equal to $Lk(\mathbf{x}, \mathbf{y})$ defined by (3) (which again is applicable to open ribbons). Second, and perhaps more fundamentally, even if that were the case, the linking $Lk(\mathbf{x}, \mathbf{y})$ is **not** an invariant to motions which change the shape of the interior of the ribbon but leave its ends fixed. Thus Lk cannot act as a topological anchor mediating changes in internal twisting for global geometry, the fundamental role of the theorem. This is crucial for example in the case of DNA supercoiling experiments where a fixed number of rotations are applied at the ribbon's ends, constraining the ribbon topology through over/under rotation. By contrast the net-winding L precisely measures this input and can be used to constrain the ribbon's topology.

To see why this is, we note that the integral (3) still represents the signed area covered on the unit sphere by the map $\mathscr{C}(s,t)$. However, this is no-longer an integer and has a boundary represented by all the chords $\mathscr{C}(0,t)\ \forall t \in [0, L_2]$, $\mathscr{C}(L_1,t)\ \forall t \in [0, L_2]$, $\mathscr{C}(s,0)\ \forall s \in [0, L_1]$ and $\mathscr{C}(s, L_2)\ \forall s \in [0, L_1]$. Since this boundary depends on the geometry of the interior of the curves $\mathbf{x}$ and $\mathbf{y}$ it can change even if the boundary points stay fixed. Another way of putting this is in terms of link projections. As in the closed ribbon case, if we project the link along a direction $\mathbf{n}$ and count crossings we can define a planar linking. $Lk(\mathbf{x}, \mathbf{y})$ is again the average over all such projections. However, now, the planar linking will not be the same for all directions and specific projections can lose crossings if the ribbon's interior deforms to pass under/above one of its end points in projection as indicated in Figure 9(b).

Since the net-winding only measures the crossing of chords $C_z(z)$, the only contributions involving the end points occur **between** the end points, *i.e.* the angles $\Theta(x(0), y(0))$ and $\Theta(x(L), y(L))$ which lie in the planes shown in Figure 9(a). Thus if they are fixed, there is no change to the boundary of the calculation (see Theorem 4 of[12]). Put simpler: the projection average involved in the net winding will not include the possibility of the interior crossing the ribbon's end, as is the case in 9(b).

Knot disentanglement revisited

We recall the knot disentanglement deformation depicted in Figure 10 of the main paper and whose writhe calculations are shown in Figure 12 of the main paper. The first tranche of the knot's deformation covers the loosening of the knot. This is achieved by extending the end of the knot with a straight line as shown in Figure 10 (a procedure which produces the transition from Figure 10 (a) to (b) of the main text). Since the writhe is invariant under a uniform scaling, this is equivalent to pulling the end of the knot tight (in reverse in the calculations). Thus the shape of the knotted section is unaltered. The calculations of the Wr (defined by (7)) for this curve show a steady change in value where the W_p calculations do not. This occurs because the Wr calculation involves an average over directions which see the development of additional crossings of the curves interior and its end point, as shown in Figure 10(a) and (b). By contrast the W_p calculation is an average over projection directions which do not see these extra crossings, see Figures 10(c) and (d).

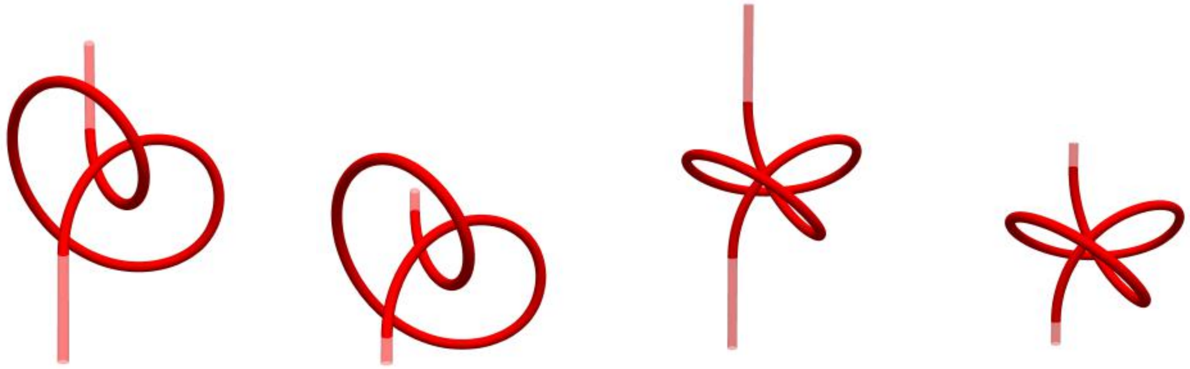

(a) **(b)**

Figure 10. Points of view of a pair of curves from the set whose writhe values are shown in Figure 12 of the main text. The opaque sections of curve are the extensions used to create a pulled tight knot as shown in Figure 10(a) of the main paper. Panels (a) and (b) indicate the projection from which a planar writhe calculation *wr* could be made which would be part of the average involved in Wr, equation (7), but **not** W_p. As the knot is "loosened" (the extension shrunk), a crossing present in panel (a) disappears in (b). Panels (c) and (d) are the same curves shown in (a) and (b) respectively, but in this case the projection from which a planar writhe calculation *wr* could be made would be part of the average involved both W_r **and** W_p. There is no loss/gain in crossings.

Closures

Approaches such as those outlined by[3,13,15,18,19] discuss methods of artificial closure of the ribbon such that an invariant link measure is recovered and the original closed Călugăreanu theorem is valid. As an example, work by Starostin[20] highlights a possible extension through closure of the unit tangent curve on the surface of the unit sphere. It was shown in[12] that it is always possible to extend an open curve $\mathbf{x}$ with a section of curve $\mathbf{x}_c$ to form a closed curve $\mathbf{x}\cup\mathbf{x}_c$, such that $W_p(\mathbf{x}) = W_r(\mathbf{x}\cup\mathbf{x}_c)$. In[13] it was further shown that it is always possible to extend a ribbon in a similar manner such that $L(\mathbf{x},\mathbf{y}) = Lk(\mathbf{x}\cup\mathbf{x}_c,\mathbf{y}\cup\mathbf{y}_c)$. However, we stress that from the point of view of topologically constraining the ribbon structure, the development of a closure is an unnecessary complication given that the net winding provides the necessary topological constraint without closure. In addition, as we have highlighted, the polar writhe/net winding formulation of this constraint has the additional benefit of a clear local/non-local decomposition.

The Fuller writhe of open curves

Several authors suggested the following expression for quantifying the writhing of an open curve:

$$W_F(\mathbf{x}) = \int_0^L \frac{\hat{z}\cdot\mathbf{T}\times\frac{\mathrm{d}\mathbf{T}}{\mathrm{d}s}}{1+\hat{z}\cdot\mathbf{T}}\mathrm{d}s. \tag{17}$$

This is nearly identical to the expression for W_{pl} without the absolute argument in the denominator ($\hat{z}\cdot\mathbf{T}$ not $|\hat{z}\cdot\mathbf{T}|$). The quantity in (17) is labelled W_F here as it can be derived from the Fuller ΔW_r expression (equation 14)[3]. It was also independently derived in[4] and[5] as a limited means of topologically constraining the molecule using descriptions of the curve $\mathbf{x}$ in terms of the three-dimensional group of rotations. However, as demonstrated in[18], it cannot be used to fully topologically constrain the structure (in particular in plectomemed configurations). This can be made clear in its relationship to the polar writhe. It was shown in[12] that $W_F(\mathbf{x})$ given by (17) is equal to $W_p^{\dagger}(\mathbf{x})$ given by (15). This makes clear that the missing contribution is the integer windings of each pair of distinct sections of the curve $\mathbf{x}_i$ and thus with the WASP package it would be possible to precisely quantify the error in the expression (17).

References

1. Calugareanu, G. L'intégrale de gauss et l'analyse des nœuds tridimensionnels. *Rev. Math. pures appl* **4** (1959).

2. Fuller, F. B. Decomposition of the linking number of a closed ribbon: A problem from molecular biology. *Proc Natl Acad Sci U S A* **75**, 3557–3561 (1978). 16592550[pmid].
3. Rossetto, V. & Maggs, A. C. Writhing geometry of open dna. *The J. Chem. Phys.* **118**, 9864–9874, DOI: 10.1063/1.1569905 (2003).
4. Bouchiat, C. & Mézard, M. Elasticity model of a supercoiled dna molecule. *Phys. Rev. Lett.* **80**, 1556–1559, DOI: 10.1103/PhysRevLett.80.1556 (1998).
5. Fain, B., Rudnick, J. & Östlund, S. Conformations of linear dna. *Phys. Rev. E* **55**, 7364–7368, DOI: 10.1103/PhysRevE.55.7364 (1997).
6. Samuel, J., Sinha, S. & Ghosh, A. Dna elasticity: topology of self-avoidance. *J. Physics: Condens. Matter* **18**, S253 (2006).
7. Van der Heijden, G. & Thompson, J. Helical and localised buckling in twisted rods: a unified analysis of the symmetric case. *Nonlinear dynamics* **21**, 71–99 (2000).
8. Ricca, R. L. & Nipoti, B. Gauss'linking number revisited. *J. Knot Theory Its Ramifications* **20**, 1325–1343 (2011).
9. Dennis, M. & Hannay, J. Geometry of călugăreanu's theorem. *Proc. Royal Soc. Lond. A: Math. Phys. Eng. Sci.* **461**, 3245–3254, DOI: 10.1098/rspa.2005.1527 (2005). http://rspa.royalsocietypublishing.org/content/461/2062/3245.full.pdf.
10. Frankel, T. *The geometry of physics: an introduction* (Cambridge university press, 2011).
11. Banchoff, T. Self linking numbers of space polygons. *Indiana Univ. Math. J.* **25**, 1171–1188 (1976).
12. Berger, M. A. & Prior, C. The writhe of open and closed curves. *J. Phys. A: Math. Gen.* **39**, 8321 (2006).
13. Prior, C. B. & Neukirch, S. The extended polar writhe: a tool for open curves mechanics. *J. Phys. A: Math. Theor.* **49**, 215201 (2016).
14. Klenin, K. & Langowski, J. Computation of writhe in modeling of supercoiled dna. *Biopolym. Orig. Res. on Biomol.* **54**, 307–317 (2000).
15. Agarwal, P. K., Edelsbrunner, H. & Wang, Y. Computing the writhing number of a polygonal knot. *Discret. & Comput. Geom.* **32**, 37–53 (2004).
16. White, J. H. Self-linking and the gauss integral in higher dimensions. *Am. J. Math.* **91**, 693–728 (1969).
17. Aldinger, J., Klapper, I. & Tabor, M. Formulae for the calculation and estimation of writhe. *J. Knot theory its Ramifications* **4**, 343–372 (1995).
18. Neukirch, S. & Starostin, E. L. Writhe formulas and antipodal points in plectonemic dna configurations. *Phys. Rev. E* **78**, 041912, DOI: 10.1103/PhysRevE.78.041912 (2008).
19. Patil, V. P., Sandt, J. D., Kolle, M. & Dunkel, J. Topological mechanics of knots and tangles. *Science* **367**, 71–75 (2020).
20. Starostin, E. On the writhing number of a non-closed curve. In *Physical And Numerical Models In Knot Theory: Including Applications to the Life Sciences*, 525–545 (World Scientific, 2005).
